# A multi-trait systems approach reveals a response cascade to bleaching in corals

**DOI:** 10.1186/s12915-017-0459-2

**Published:** 2017-12-07

**Authors:** Stephanie G. Gardner, Jean-Baptiste Raina, Matthew R. Nitschke, Daniel A. Nielsen, Michael Stat, Cherie A. Motti, Peter J. Ralph, Katherina Petrou

**Affiliations:** 1 Climate Change Cluster, University of Technology Sydney, Ultimo, 2007 NSW Australia; 2 School of Life Sciences, University of Technology Sydney, Ultimo, 2007 NSW Australia; 3 Centre for Environmental and Marine Studies (CESAM), University of Aveiro, 3810-193 Aveiro, Portugal; 4 Trace and Environmental DNA (TrEnD) Laboratory, Department of Environment and Agriculture, Curtin University, Perth, 6102 WA Australia; 5 Australian Institute of Marine Science, Townsville, 4810 QLD Australia

**Keywords:** Coral bleaching, Reactive oxygen species, Antioxidants, Dimethylsulfoniopropionate, *Acropora millepora*, *Stylophora pistillata*

## Abstract

**Background:**

Climate change causes the breakdown of the symbiotic relationships between reef-building corals and their photosynthetic symbionts (genus *Symbiodinium*), with thermal anomalies in 2015–2016 triggering the most widespread mass coral bleaching on record and unprecedented mortality on the Great Barrier Reef. Targeted studies using specific coral stress indicators have highlighted the complexity of the physiological processes occurring during thermal stress, but have been unable to provide a clear mechanistic understanding of coral bleaching.

**Results:**

Here, we present an extensive multi-trait-based study in which we compare the thermal stress responses of two phylogenetically distinct and widely distributed coral species, *Acropora millepora* and *Stylophora pistillata*, integrating 14 individual stress indicators over time across a simulated thermal anomaly. We found that key stress responses were conserved across both taxa, with the loss of symbionts and the activation of antioxidant mechanisms occurring well before collapse of the physiological parameters, including gross oxygen production and chlorophyll *a.* Our study also revealed species-specific traits, including differences in the timing of antioxidant regulation, as well as drastic differences in the production of the sulfur compound dimethylsulfoniopropionate during bleaching. Indeed, the concentration of this antioxidant increased two-fold in *A. millepora* after the corals started to bleach, while it decreased 70% in *S. pistillata*.

**Conclusions:**

We identify a well-defined cascading response to thermal stress, demarking clear pathophysiological reactions conserved across the two species, which might be central to fully understanding the mechanisms triggering thermally induced coral bleaching. These results highlight that bleaching is a conserved mechanism, but specific adaptations linked to the coral’s antioxidant capacity drive differences in the sensitivity and thus tolerance of each coral species to thermal stress.

**Electronic supplementary material:**

The online version of this article (doi:10.1186/s12915-017-0459-2) contains supplementary material, which is available to authorized users.

## Background

Coral reefs are the most biodiverse and productive marine ecosystems on the planet [1] and their ecological success can largely be attributed to the symbiosis that corals form with dinoflagellates from the genus *Symbiodinium* [2]. Despite their ecological importance and persistence over geological time, coral reefs are one of the most vulnerable marine ecosystems in a changing climate [3–5]. Seawater temperatures above monthly and annual averages have been linked to cellular damage in the algal symbionts and coral host, leading to the breakdown of the symbiosis and subsequent expulsion or loss of symbionts from the host, a process termed coral bleaching [6].

Early attempts to characterise the cellular mechanisms behind coral bleaching focused primarily on the physiology of the symbiont [7], where stress-induced impairment of photosynthesis was demonstrated to increase formation of reactive oxygen species (ROS), causing further damage to the photosynthetic machinery [6, 8, 9]. As such, historically, coral bleaching has chiefly been described as a result of the symbiont response to stressors [7, 10, 11]. With the advance in molecular techniques, it became apparent that corals can form symbioses with multiple *Symbiodinium* clades [12–14], some of which are more thermally tolerant than others [15, 16], which has proved to be of significance to the overall sensitivity of a coral to bleaching conditions. Nonetheless, bleaching susceptibility differs widely among different coral genera despite often hosting the same *Symbiodinium* clade [17, 18] as well among individual corals hosting the same symbiont type [17–19], suggesting that host physiology plays a key role in the bleaching process.

Bleaching susceptibility in reef-building corals is not well modelled due to the difficulties of understanding the tolerances of the individual components of the holobiont, comprising the cnidarian host, its symbiotic algae and microbes [20]. Yet, it is widely agreed that oxidative stress in the symbiont and/or host plays a key role in the breakdown of the symbiotic partnership [21, 22]. Highly conserved enzymatic and non-enzymatic antioxidant systems operate in both the host and the symbiont simultaneously and, thus, accurately describing the localised antioxidant response is challenging. In corals, key antioxidants are essential for maintaining cell homeostasis and modulating stress-induced ROS in the cell. These include superoxide dismutase (SOD), an endogenous antioxidant which reduces the damaging potential of superoxide anion radicals (O_2_^•-^) by catalysing its dismutation to hydrogen peroxide (H_2_O_2_); catalase (CAT and CAT-like activity), which subsequently detoxifies H_2_O_2_ converting it into water and O_2_ along with peroxidases such as ascorbate peroxidase [23]; and glutathione (GSx), which is involved either directly as an antioxidant by reacting with singlet oxygen (^1^O_2_), O_2_^•-^ and hydroxyl radicals (OH^•^), or indirectly as a reduction equivalent in the regeneration of ascorbate in the ascorbate-glutathione cycle [6]. Another potential means to alleviate cellular oxidative stress involves the production of dimethylsulfoniopropionate (DMSP). This organosulfur compound, together with its breakdown products, dimethylsulfide (DMS) and acrylate, can function as an effective antioxidant system [24]. Upon reacting with ROS, DMSP and DMS are oxidised to form dimethyl sulfoxide (DMSO), which constitutes a secondary shield against ROS that can be further oxidised. In adult corals, DMSP is produced by *Symbiodinium* [25], and is also highly likely synthesised by the cnidarian host [26] and some associated bacteria [27]. The extremely high DMSP concentrations resulting from these multiple sources may function as an antioxidant in the coral holobiont.

While it remains unknown if the adaptive mechanisms used by corals are sufficient to allow them to persist under the current rate of climate change [28], the substantial inter- and intraspecific variation in thermotolerance exhibited by different species of corals suggests that there will be ‘winners’ and ‘losers’ under future climate [18]. To estimate the rate of adaptation and acclimation under a changing climate, we need to understand how corals respond to various environmental stressors through the expression of functional traits [29]. Mounting evidence suggests that the response of corals and their symbionts varies significantly in terms of timing and severity of the expression of key metabolic genes [30], but also that the cnidarian host responses precede the bleaching process and symbiont dysfunction [7, 31–33]. As such, the common focus on the response of the symbiont alone in defining coral holobiont stress severely underestimates the importance of early cellular events in the host [33].

Herein, we investigated a range of physiological and biochemical traits involved in the thermal stress-response in two phylogenetically distinct and abundant scleractinian corals, *Acropora millepora* and *Stylophora pistillata*. Fourteen physiological parameters were measured to assess the responses of the host and symbiont components, including stress-response metabolites involved in quenching of ROS (SOD, CAT-like activity and GSx), vitality indicators such as photosynthesis and respiration, as well as intracellular DMSP and DMSO. Our results reveal time-dependent key thermal stress-responses that are conserved across the two taxa, as well as species-specific responses and tolerance to bleaching as a result of oxidative stress.

## Methods

### Species collection

Colonies of *A. millepora* and *S. pistillata* (n = 5 of each) were collected from Heron Island lagoon (<2 m depth at low tide, collected a minimum of 20 m apart) in the southern Great Barrier Reef, Australia (152° 06′E, 20° 29′S), in February 2015 (Australian summer). Coral colonies were split in half, and divided between the control and treatment groups before being broken into fragments (~3 cm each) and fixed onto glass slides using Selley’s Epoxy (Selleys Pty Ltd, Australia). Coral fragments were left to acclimate for 5 days in a flow-through aquarium system under 50% shading (max 700 μmol photons m^-2^ s^-1^ under water) and ambient temperature (27 ± 0.5 °C).

### Experimental set-up

Ten experimental tanks (10 coral fragments of each species per 90 L tank) were set up in shaded, semi-closed, recirculating flow through aquaria (flow rate ~1 L/min) with a constant flow of lagoon seawater (~27 ± 0.5 °C). The ambient light intensity was measured every 5 min using Odyssey PAR loggers (Dataflow Systems Limited, Christchurch) and temperature was recorded every 10 min with temperature sensors (Thermochron, Australia). For the thermal stress treatment tanks (n = 5), the temperature was increased by 1 °C per day from 27 °C (day 1) to 30 °C (day 4), then by 0.5 °C per day to reach the target temperature of 32 °C (day 8) and then held (excluding some diel fluctuations; Additional file 1: Figure S1) for a further 7 days (*S. pistillata*) or 10 days (*A. millepora*), during which sub-sampling was conducted every 3 days (equating to a total of four time-points for *S. pistillata*, and five for *A. millepora*). Using a pulse amplitude-modulated (PAM) fluorometer (Mini PAM, Walz GmbH, Effeltrich, Germany; MI: 12, Gain: 12, SI: 12, SW: 0.8 s) chlorophyll *a* fluorescence was measured every midday (effective quantum yield of photosystem II (PSII); ΔF/F_M_') and just after sunset (maximum quantum yield of PSII; F_V_/F_M_) to monitor photophysiological stress throughout the experiment. At each sampling time-point, fluorescence steady state light curves (SSLC) and gross photosynthesis/respiration were measured on individual fragments (see below for detail). Fragments were also collected, snap frozen in liquid N_2_ and stored at −80 °C for *Symbiodinium* diversity and antioxidant assays (SOD, GSx, CAT and CAT-like activity). Another set of fragments were stored in methanol at −20 °C for DMSP/DMSO determination using quantitative ^1^H nuclear magnetic resonance (qNMR) spectroscopy.

### Chlorophyll *a* fluorescence

Photosynthetic efficiency of the algal symbionts in *A. millepora* and *S. pistillata* was measured via chlorophyll *a* fluorescence using an Imaging PAM (Max/K, Walz GmbH; MI: 12, Gain: 12, SI: 12, SW: 0.8 s). At each sampling time-point, fragments (n = 5) were placed in a large shallow beaker containing circulating, temperature-controlled seawater of the corresponding treatment and dark adapted for 15 min. Following dark adaptation, minimum fluorescence (F_O_) was recorded before application of a high intensity saturating pulse of light (saturating pulse width = 0.8 s; saturating pulse intensity > 3000 μmol photons m^-2^ s^-1^), where maximum fluorescence (F_M_) was determined. From these two parameters, the maximum quantum yield of PSII was calculated as F_V_/F_M_ = (F_M_–F_O_)/F_M_ [34]. Following F_V_/F_M_, a seven-step SSLC was conducted with each light level (56, 111, 231, 396, 531, 701 and 926 μmol photons m^-2^ s^-1^) applied for 3 min before recording the light-adapted minimum (F_T_) and maximum fluorescence (F_M_') values. The relative electron transport rates from the SSLCs were calculated according to Ralph and Gademann [35].

### Photosynthesis and respiration

Photosynthesis and respiration rates were measured on coral fragments using oxygen optodes (PyroScience GmbH, Germany) and custom-made closed chambers (80 mL). Each chamber was filled with seawater at the temperature of each respective treatment and positioned in a temperature controlled water bath (treatment temperature ± 0.5 °C). Light was supplied via white LED strips positioned around each chamber and light intensity calibrated using a 4π sensor. Oxygen optodes were connected to a FireSting O_2_ logger and data was acquired using the FireSting software (PyroScience GmbH, Aachen, Germany). The optode was calibrated according to the manufacturer’s protocol immediately prior to measurements using a freshly prepared sodium thiosulfate solution (10% w/w) and air-bubbled filtered seawater (FSW; 0.2 μm) at experimental temperatures for 0% and 100% air saturation values, respectively. Oxygen concentration was measured every 1 to 2 min in each chamber until a linear change in rate was recorded for each replicate, with measurements taken first in the dark and subsequently in the light (300 μmol photons m^-2^ s^-1^). Gross photosynthesis (ΔO_2_[light]–ΔO_2_[dark]), respiration rates and P:R ratios (light) were calculated for each treatment (n = 5) and time-point. The coral fragments were subsequently used for cell density, chlorophyll *a* and surface area calculations (methods below).

### *Symbiodinium* diversity

Next-generation sequencing was used to measure *Symbiodinium* diversity associated with the study corals [36, 37]. Total nucleic acids were extracted from independent coral fragments from the corresponding colony using the PowerPlant Pro DNA Isolation Kit (MoBio Laboratories, CA, USA) according to the manufacturer’s instructions. Amplification of target DNA was performed in a single round of polymerase chain reaction (PCR) using fusion tag primers consisting of Illumina adaptor and sequencing primers, indexes unique to this study, and the template-specific primers ITSD (5′-GTGAATTGCAGAACTCCGTG-′3) and ITS2rev2 (5′-CCTCCGCTTACTTATATGCTT-′3) that target the partial 5.8S, entire ITS2, and partial 28S region of *Symbiodinium* [38]. For PCR preparation and conditions as well as amplicon library preparation, see supporting information.

Sequences were assembled using the Illumina MiSeq software under default settings and then passed through a series of quality control steps prior to assignment into operational taxonomic units (OTU) and blastn analyses. For details on sequence downstream analyses and identification please see supporting information. NGS data are available in Figshare (doi:10.6084/m9.figshare.5596810) and representative OTU sequences are available in GenBank (Accession numbers: KY825747-KY825767).

### Cell density, chlorophyll *a* and surface area

Coral tissue was removed from the skeleton using air blasting in 10 mL FSW (0.2 μm) [39]. The tissue slurry was concentrated via centrifugation at 1000 *g* for 10 min. The algal pellets were resuspended in 5 mL FSW and homogenised. A 2-mL aliquot was then centrifuged at 3600 *g* for 4 min, the supernatant removed and the pellet resuspended in 3 mL of 90% acetone and left at 4 °C in the dark for 24 h. Following extraction, the sample was re-centrifuged at 3600 *g* for 4 min and the supernatant used for spectrophotometric chlorophyll *a* determination (using 664 nm wavelength), following the equation from Ritchie [40]. An additional 1 mL aliquot of the coral homogenate was resuspended in 1 mL phosphate-buffered saline: paraformaldehyde (PBS:PFA) solution and stored at room temperature for cell density measurements using a haemocytometer (n = 8 per replicate). The tissue-free coral skeletons were bleached in a 10% bleach solution for 24 h to remove residual organic materials [41, 42] and oven dried before the surface area of each coral fragment was determined using the paraffin wax technique [42, 43].

### Antioxidant and enzyme activity

Coral tissues from *A. millepora* and *S. pistillata* were extracted from snap-frozen fragments in 5 mL FSW (0.2 μm) using air blasting and the homogenate was concentrated by centrifugation at 3600 *g* for 10 min at 4 °C. The supernatant was transferred to a clean Falcon tube and assays were run immediately on the host tissue extract, while the algal pellet was frozen at −80 °C until further analysis. To minimise host cell contamination in symbiont enzyme assays, prior to further processing, algal pellets were resuspended in 2 mL FSW, centrifuged at 3600 *g* for 10 min at 4 °C, and the supernatant discarded. This was repeated twice before cells were ruptured via sonication (3 × 10 s pulse on ice; Vibra Cell VC50T, Sonics & Materials, USA). The suspension of lysed cells was centrifuged at 3600 *g* for 10 min at 4 °C and the supernatant used for antioxidant assays and to determine total protein. Assays were run using the specific reaction buffer provided by each assay kit (SOD, GSx, CAT and total protein), according to the manufacturers’ guidelines (refer to kit protocols for details). Total protein was measured using the Pierce™ BCA Protein Assay Kit (Thermo Scientific, USA) after incubation at 37 °C for 45 min, for host and algal components (individually). Because host and symbiont protein content did not change with treatment, all enzyme results (SOD, GSx and CAT) are expressed in specific activities (U mg^-1^ protein).

Total SOD (including Cu/Zn and Mn isoforms) was determined in triplicate per sample using a superoxide dismutase activity colorimetric determination kit (SOD-560, Applied Bioanalytical Labs) and absorbance measured at 560 nm with a FLUOstar OPTIMA plate reader (BMG Labtech, Germany) at room temperature (21 °C). Results are expressed in the ‘standard cytochrome c’ SOD unit (U), by measuring the ratios of auto-oxidation rates in the presence and absence of the sample. Total GSx was measured using a glutathione colorimetric assay kit (CS0260, Sigma Aldrich), and the yellow product, nitrobenzoic acid, was measured spectrophotometrically at 412 nm with a FLUOstar OPTIMA plate reader (BMG Labtech, Germany) at room temperature. Catalase activity was determined using a catalase fluorometric detection kit (ADI-907-027, Enzo Life Sciences) with colour reaction assessed according to the manufacturer at excitation 530–570 nm and emission 590–600 nm (TECAN Infinite® 200 PRO microplate reader, Switzerland) at room temperature.

### Quantification of DMSP/DMSO concentrations

Concentrations of DMSP/DMSO were determined using qNMR spectroscopy. Sample extractions were based on modified methods from Tapiolas et al. [44]. Coral fragments were stored in 3 mL of methanol at −20 °C until processing. Immediately prior to processing, coral fragments were extracted a second time with 1 mL of HPLC-grade methanol (CH_3_OH) for 1 min with sonication on ice. The two extracts were pooled and dried using a concentrator (Savant SpeedVac, Thermo Scientific, USA). The dried extracts were resuspended in a mixture of deuterated methanol (CD_3_OD; 750 μL) and deuterium oxide (D_2_O; 250 μL), vortexed to solubilise the compounds and then centrifuged to pellet the debris. A 700-μL aliquot of the particulate-free extract was transferred into a 5-mm Norell 509-UP NMR tube and analysed immediately by ^1^H qNMR. Spectra were recorded on a Bruker Avance 600 MHz NMR spectrometer (Bruker, Germany) with a TXI cryoprobe, referenced using CD_3_OD (δ_H_ 3.31). Spectra were acquired following the methods in Tapiolas et al. [44]. After calibration, the concentrations of DMSP and DMSO in the NMR sample were determined by comparing the signal integrals of well resolved non-exchangeable protons (**CH**_**3**_)_2_SCH_2_CH_2_CO_2_ centred at δ 2.95 ppm for DMSP and (**CH**_**3**_)_2_SO δ 2.73 ppm for DMSO in a 0.20 ppm window against the electric reference signal [45]. Quantification was performed using the ERETIC method (Electronic REference To access In vivo Concentrations) [45]. This technique electronically generates an external reference signal during the data acquisition that is calibrated using stock solutions of 4 mM acrylate and DMSP [41, 44] based on the concentration present in test samples. DMS is soluble in methanol [46] but oxidises into DMSO over time in coral extracts [44]. Therefore, the DMSO signals measured here can be considered as a proxy for the combined DMS and DMSO (DMS/DMSO) pools. The surface area of the remaining coral skeleton was measured (as described above) and the DMSP/DMSO data were normalised to both coral surface area and *Symbiodinium* density.

### Statistical analysis

All physiological variables were analysed by a two-factor univariate PERMANOVA, using a resemblance matrix based on Euclidean distance and factors as fixed effects. The PERMANOVA was run using 9999 permutations to obtain *P* values using the Monte Carlo method. Analyses were carried out using Primer v.6 statistical package [47] in conjunction with the PERMANOVA+ module [48]. The majority of variables showed a significant interaction term between temperature and time (Additional file 1: Table S1, S2), indicating that most data showed a temporal pattern that was dependent on the temperature treatment. Since significant interaction terms often indicate that the test for main effects is not very informative [49], pair-wise comparison PERMANOVA tests were conducted for temperature at each time level and for time at each temperature level, utilising the Monte Carlo method. Significant differences in pair-wise comparisons at each time point are denoted with asterisks, while significant changes within a treatment over time is denoted by superscript lettering. Unless otherwise stated, all statistical results reported are from the PERMANOVA analysis. An independent samples *t* test was used to determine differences between species for the relative changes at the final time-point in the thermal treatment (Fig. 4 and Additional file 1: Figure S9) using IBM SPSS Statistics v.21 (IBM Corporation, New York). The frequency of *Symbiodinium* OTUs that occurred in each sample was first standardised and square-root transformed, then used to generate a Bray–Curtis dissimilarity matrix and one-way ANOSIM with species as a factor in PRIMER v.6 [47], and a heatmap using the gplots package in R [50]. Averaged values are reported as mean ± standard error (SE) throughout, unless otherwise stated.

## Results

### Thermal stress leads to a decline in host and symbiont health

The *Symbiodinium* diversity was significantly different between the two coral species (ANOSIM test; R = 1, *P* < 0.05). A total of 21 OTUs were present in the samples (20 clade C and 1 clade B); 10 of these clade C OTUs were associated with *A. millepora*, while 12 clade C and 1 clade B were associated with *S. pistillata*. In *A. millepora*, C_OTU1_ (represented by an ITS2 sequence with 98.9% similarity to C3) had a relative abundance of 98.5% (Additional file 1: Figure S2). *S. pistillata* was associated with a distinct clade C OTU (C_OTU2_ identical to C8a; Additional file 1: Figure S2), which represented 86.1% of sequences.

**Fig. 1:**
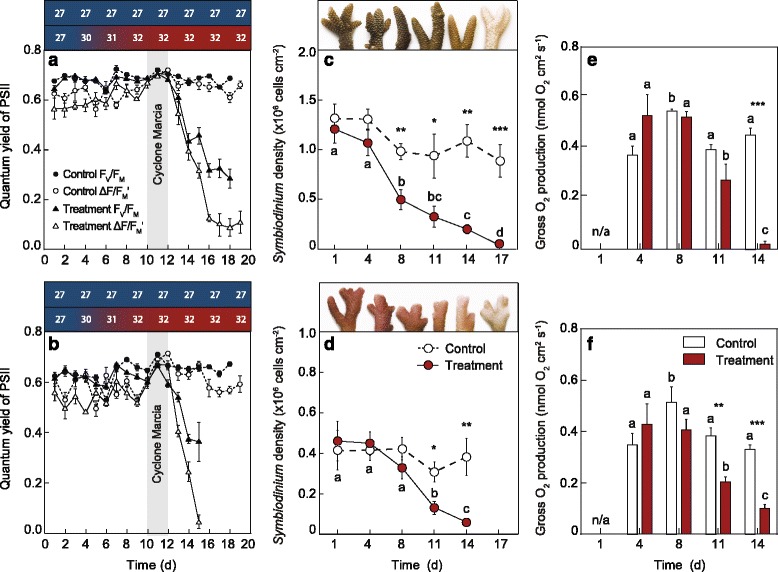
Physiological parameters for (**a**) *Acropora millepora* and (**b**) *Stylophora pistillata* showing effective quantum yield of photosystem (PS)II (ΔF/F_M_'; white symbols) and maximum quantum yield of PSII (F_V_/F_M_; black symbols) for the controls (27 °C; circle symbols) and treatments (32 °C; triangle symbols). *Symbiodinium* cell density for (**c**) *A. millepora* and (**d**) *S. pistillata* for the control (27 °C; white circles, dashed line) and treatment (32 °C; red circles, solid line) with images of coral fragments from each time-point showing visual paling over time*.* The gross oxygen production for (**e**) *A. millepora* and (**f**) *S. pistillata* for the control (27 °C; white bars) and treatment (32 °C; red bars) at each time point (n = 4). Asterisks indicate significant differences between treatments where *P < 0.05, ** *P* < 0.01 and ***P < 0.001, and letters indicate significant differences between time for the heat-treated samples at *P* < 0.05 (n = 5). Averages (± SE) shown for all

Colony fragments of *A. millepora* and *S. pistillata* exposed to thermal stress showed clear signs of bleaching (i.e. visual paling) over the course of the study. Exposure to elevated temperature (32 °C) caused a significant decline in the maximum quantum yield of PSII (F_V_/F_M_) by day 13 for both species (*A. millepora*, pseudo F_17,179_ = 43.88, *P*_MC_ = 0.0001; *S. pistillata*, pseudo F_17,179_ = 73.39, *P*_MC_ = 0.0001; Fig. 1a, b). In *A. millepora*, there was an 84% decrease in the effective quantum yield of PSII (ΔF/F_M_') from 0.66 ± 0.02 on day 1 to 0.11 ± 0.05 on day 19 (Fig. 1a). There was an even more rapid decline observed in *S. pistillata*, with an 89% decrease from 0.58 ± 0.04 on day 1 to 0.06 ± 0.03 on day 15 (Fig. 1b). The two-day interference of cyclone Marcia at Heron Island between days 10 and 12 brought increased cloud cover and decreased irradiance, temporarily increasing ΔF/F_M_' and F_V_/F_M_ in the treatment. However, once passed, ΔF/F_M_' and F_V_/F_M_ declined from day 12 to 19 for *A. millepora* and day 12 to 15 for *S. pistillata*.

Steady state light curves showed a decline in the relative electron transport rates under elevated temperature for both species by day 11 (Additional file 1: Figure S3) with further declines by day 14 (Additional file 1: Figure S3b, d). *Symbiodinium* cell density decreased, with significant differences between treatments from day 8 in *A. millepora* (pseudo F_5,59_ = 3.04, *P*_MC_ = 0.019; Fig. 1c) and day 11 for *S. pistillata* (pseudo F_4,49_ = 2.78, *P*_MC_ = 0.038; Fig. 1d). By day 14, both species exposed to thermal stress had lost more than 80% of their initial cell density (Fig. 1c, d) and, while *A. millepora* showed an earlier onset of decline in cell density, *Symbiodinium* density was < 5% by day 17, whereas in *S. pistillata*, a similar cell loss (> 95%) had occurred 3 days earlier (day 14). There were also significant declines in chlorophyll *a* for *A. millepora* (pseudo F_5,59_ = 4.10, *P*_MC_ = 0.005; Additional file 1: Figure S4a) and *S. pistillata* by day 11 (pseudo F_4,49_ = 5.83, *P*_MC_ = 0.001; Additional file 1: Figure S4b).

Gross oxygen production declined significantly in heat-treated *A. millepora* (pseudo F_3,31_ = 17.58, *P*_MC_ = 0.0001), with decreased production by day 14 (Fig. 1e). In *S. pistillata*, gross oxygen production was significantly lower than the control by days 11 and 14 in the heat-treated corals (pseudo F_3,31_ = 4.88, *P*_MC_ = 0.0087; Fig. 1f). O_2_ flux in the light (net photosynthesis) differed over time and treatment in *A. millepora* (pseudo F_3,31_ = 18.70, *P*_MC_ = 0.0001; Additional file 1: Figure S5a) and *S. pistillata* (pseudo F_3,31_ = 8.12, *P*_MC_ = 0.0008; Additional file 1: Figure S5b), with significant decreases in O_2_ flux detected on days 11 and 14 (Additional file 1: Figure S5a, b). There was a significant effect of treatment and time in the respiration rate for *A. millepora* (pseudo F_3,31_ = 6.77, *P*_MC_ = 0.002), with increased rates measured in the heat-treated corals on day 8 (Additional file 1: Figure S5a). Respiration rates remained fairly constant in *S. pistillata*, although a significant decline from day 4 was detected on days 11 and 14 in the heat-treated corals (Additional file 1: Figure S5b). There were significant declines in the P:R ratios with elevated temperature for both species (Additional file 1: Figure S5c, d). In *A. millepora*, treatment effects were significant at day 8 and 14, while in *S. pistillata*, treatment effects became apparent on days 11 and 14. In both species, the P:R ratio dropped below 1 by day 14, indicating that the coral became net heterotrophic at the light level used (300 μmol photons m^-2^s^-1^), supporting the reduced photochemical efficiency measured by the PAM data. There was a temporal difference in significant *Symbiodinium* loss (day 8) and gross photosynthesis (day 11) in *A. millepora*, which can be explained by increased self-shading. High *Symbiodinium* cell density results in lower photosynthetic efficiency, as seen in the relationship between oxygen production and *Symbiodinium* density (Additional file 1: Figure S5e). This is congruent with initial cell densities of *A. millepora* being twice that of *S. pistillata* (Fig. 1c, d).

### DMSP concentration as a bleaching response trait is species-specific

**Fig. 2:**
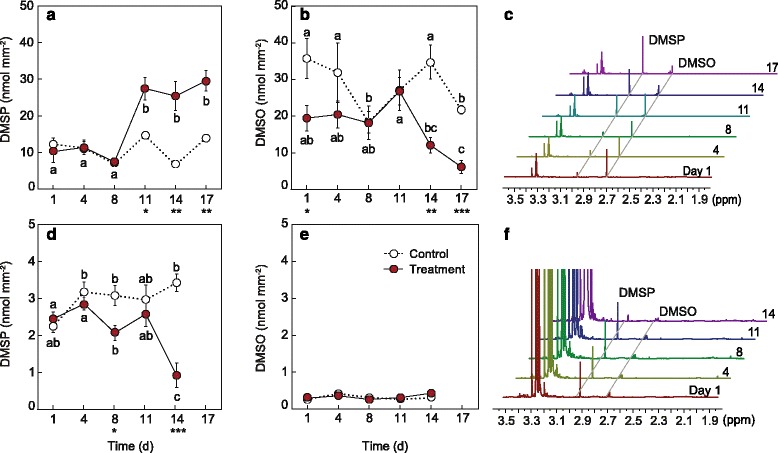
Concentrations of dimethylsulfoniopropionate (DMSP), dimethylsulfoxide (DMSO) and representative ^1^H NMR spectra for (**a**–**c**) *Acropora millepora* and (**d**–**f**) *Stylophora pistillata* for the control (27 °C; white circles, dashed line) and treatments (32 °C; red circles, solid line) over time (days). Asterisks indicate significant differences between treatments at a given time point where *P < 0.05, **P < 0.01 and ***P < 0.001, and letters indicate significant differences between time-points for the heat-treated samples at *P <* 0.05. Averages (± SE) shown (n = 5)

The initial concentrations of DMSP in *A. millepora* were approximately five times higher than that of *S. pistillata* (Fig. 2). There was a significant effect of time and treatment for DMSP in *A. millepora* (pseudo F_5,59_ = 31.69, *P* = 0.0001) with a sustained increase in DMSP (10.23 ± 2.93 to 29.53 ± 2.79 nmol/mm^2^) from day 11 under heat treatment (Fig. 2a). The DMSP data for *S. pistillata* increased initially in the control treatment, while there was a significant decline from 2.24 ± 0.15 to 0.92 ± 0.33 nmol/mm^2^ in the heat-treated corals by day 14 (pseudo F_4,49_ = 7.66, *P* = 0.0002; Fig. 2d).

In contrast, DMSO (as a proxy for the combined DMS/DMSO pool) decreased in *A. millepora* from 19.23 ± 2.93 to 6.05 ± 2.79 nmol/mm^2^ (Fig. 2b), with a significant difference detected between treatments from day 14 (pseudo F_1,59_ = 19.54, *P*_MC_ = 0.0002; Fig. 2b). No significant change was detected for *S. pistillata* (Fig. 2e). As with DMSP, we found higher initial concentrations of DMSO in *A. millepora* (35.29 ± 5.35 nmol/mm^2^; Fig. 2b) compared to *S. pistillata* (0.26 ± 0.05 nmol/mm^2^; Fig. 2e). When concentrations of DMSP and DMSO were normalised to *Symbiodinium* density, there was a significant increase in DMSP in both species under thermal stress (*A. millepora*, pseudo F_5,59_ = 10.70, *P*_MC_ = 0.0001; *S. pistillata*, pseudo F_4,49_ = 4.12, *P*_MC_ = 0.008), with significant differences detected by day 11 (Additional file 1: Figure S6a, c). Similarly, DMSO per cell increased significantly in both species (*A. millepora*, pseudo F_5,59_ = 4.46, *P*_MC_ = 0.0027; *S. pistillata*, pseudo F_4,49_ = 10.68, *P*_MC_ = 0.0001) by day 11 (Additional file 1: Figure S6b, d).

### Enzymatic and non-enzymatic antioxidant response in host and symbiont

**Fig. 3:**
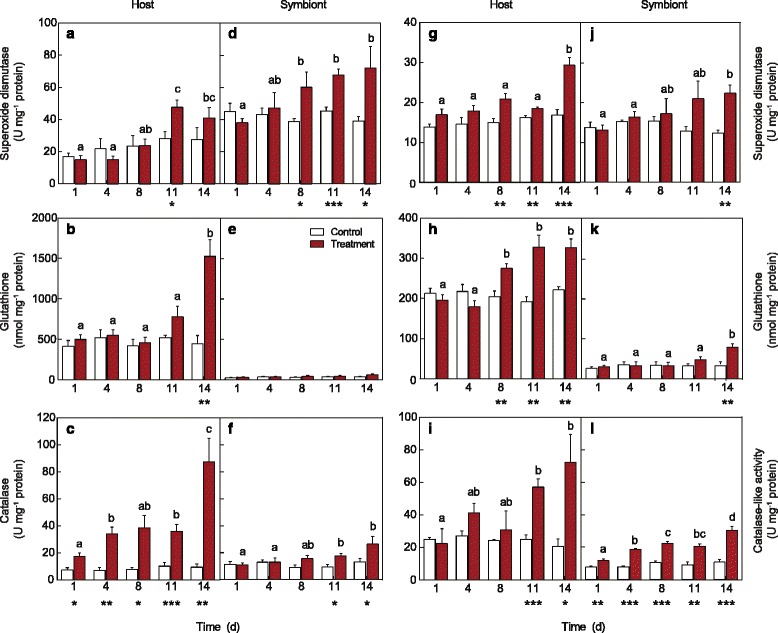
Antioxidant activity measured in the host and symbiont components of (**a**–**f**) *Acropora millepora* and (**g**–**l**) *Stylophora pistillata* for the control (27 °C; white bars) and treatment (32 °C; red bars) over 14 days, including superoxide dismutase, total glutathione and catalase (or catalase-like activity in the symbiont). Host components normalised to mg^-1^ of host protein and symbiont normalised to mg^-1^ of symbiont protein. Asterisks indicate significant differences between treatments where *P < 0.05, **P < 0.01 and ***P < 0.001, and letters indicate significant differences at *P* < 0.05. Averages (± SE) are shown (n = 4–5)

Host and symbiont antioxidant responses to thermal stress were similar for both species. There was an increase in host SOD by day 11 (pseudo F_4,49_ = 7.27, *P*_MC_ = 0.0004) and host GSx by day 14 (pseudo F_4,49_ = 8.90, *P*_MC_ = 0.0001) with elevated temperature in *A. millepora* (Fig. 3a, b). Host CAT, despite being different from control at the initial time point, increased significantly from day 1 to day 4, with an additional increase at day 14 (pseudo F_4,49_ = 7.19, *P*_MC_ = 0.0002; Fig. 3c). In thermally stressed *A. millepora* symbionts, there was a significant increase in SOD (pseudo F_4,49_ = 4.04, *P*_MC_ = 0.0089) with differences detected as early as day 8 (Fig. 3d). By day 11 there was a significant increase in CAT-like activity (pseudo F_4,49_ = 3.44, *P*_MC_ = 0.0174), which also differed significantly from the control (pseudo F_1,49_ = 10.911, *P*_MC_ = 0.0027; Fig. 3f). When normalised to cell density, significant increases in *A. millepora* symbiont CAT-like activity (pseudo F_4,49_ = 5.13, *P*_MC_ = 0.0016) were also observed by day 11 (Additional file 1: Figure S7c).

As with *A. millepora*, host antioxidant activity of thermally stressed *S. pistillata* increased significantly, including SOD (pseudo F_4,49_ = 5.53, *P*_MC_ = 0.0014; Fig. 3g), GSx (pseudo F_4,49_ = 10.02, *P*_MC_ = 0.0001; Fig. 3h) and CAT (pseudo F_4,49_ = 3.90, *P*_MC_ = 0.0095; Fig. 3i). For SOD and GSx, significant differences between treatments were detected on days 8, 11 and 14 (Fig. 3g, h); however, SOD only increased significantly by day 14. In contrast, GSx showed an earlier response, increasing on day 8 (Fig. 3h). Host CAT activity increased significantly by day 11 and remained high until day 14 (Fig. 3i). Symbiont SOD differed significantly from the control by increasing on day 14 (pseudo F_1,40_ = 9.08, *P*_MC_ = 0.0042; Fig. 3j). There were also significant changes in GSx (pseudo F_4,49_ = 3.60, *P*_MC_ = 0.0145; Fig. 3k) and CAT-like activity (pseudo F_4,49_ = 7.37, *P*_MC_ = 0.0003; Fig. 3l) for the symbionts associated with *S. pistillata*. Significant increases were detected by day 14 for symbiont GSx (Fig. 3k), while CAT-like activity increased immediately (day 4) and continued to increase, doubling by day 14 (Fig. 3l). Once normalised to *Symbiodinium* density, there were significant effects of time and treatment for all antioxidants – SOD (pseudo F_4,49_ = 7.77, *P*_MC_ = 0.0002) increased by day 14 (Additional file 1: Figure S7d), GSx (pseudo F_4,49_ = 22.12, *P*_MC_ = 0.0001) increased by day 11 (Additional file 1: Figure S7e), and CAT-like activity (pseudo F_4,49_ = 10.76, *P*_MC_ = 0.0001) was significantly greater by day 8 (Additional file 1: Figure S7f). A comparison of the total protein, normalised to surface area for host and symbiont protein, revealed no significant differences between treatments for both species, and relative changes in antioxidant concentrations could therefore be confirmed not to be a result of overall decreases in protein content (Additional file 1: Figure S8).

### Relative change in bleaching response traits over-time

**Fig. 4:**
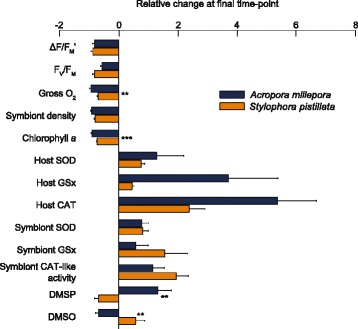
Comparison showing the relative change in measured traits from day 1 to day 14 for the host and symbiont at 32 °C, including ΔF/F_M_' (effective quantum yield of photosystem II), F_V_/F_M_ (maximum quantum yield of photosystem II), gross oxygen production, symbiont density, chlorophyll *a*, host and symbiont superoxide dismutase (SOD), glutathione (GSx), catalase (CAT; and symbiont catalase-like activity), dimethylsulfoniopropionate (DMSP) and dimethylsulfoxide (DMSO) in *Acropora millepora* (blue bars) and *Stylophora pistillata* (orange bars). Calculated as the relative change at the final time-point between the control and the treatment (X_treatment_ – X_control_/X_control_). Asterisks indicate significant differences between species at **P < 0.01 and ***P < 0.001.

Significant differences in the relative change in physiological parameters between species were detected for Gross O_2_ production (Independent samples *t* test, *t*_(8)_ = 4.78, *P* = 0.003), and chlorophyll *a* (*t*_(10)_ = 6.52, *P* = 0.0002). Additionally, there was a significant difference in the relative change in DMSP and DMSO between *A. millepora* and *S. pistillata* (*t*_(10)_ = 4.27, *P* = 0.003 and *t*_(10)_ = 3.83, *P* = 0.005, respectively; Fig. 4), where DMSP increased by 130% compared with the initial concentration in *A. millepora*, but decreased by 70% for *S. pistillata*. This contrasted with a 70% decrease in DMSO from the initial concentrations in *A. millepora* and a 58% increase in *S. pistillata* (Fig. 4). Data normalised to *Symbiodinium* cell density (Additional file 1: Figure S9) showed a significant difference between species for symbiont SOD per cell (*t*_(9)_ = 2.92, *P* = 0.019) and DMSP per cell (*t*_(10)_ = 3.47, *P* = 0.009; Additional file 1: Figure S9).

### A cascade response towards the bleached state

**Fig. 5:**
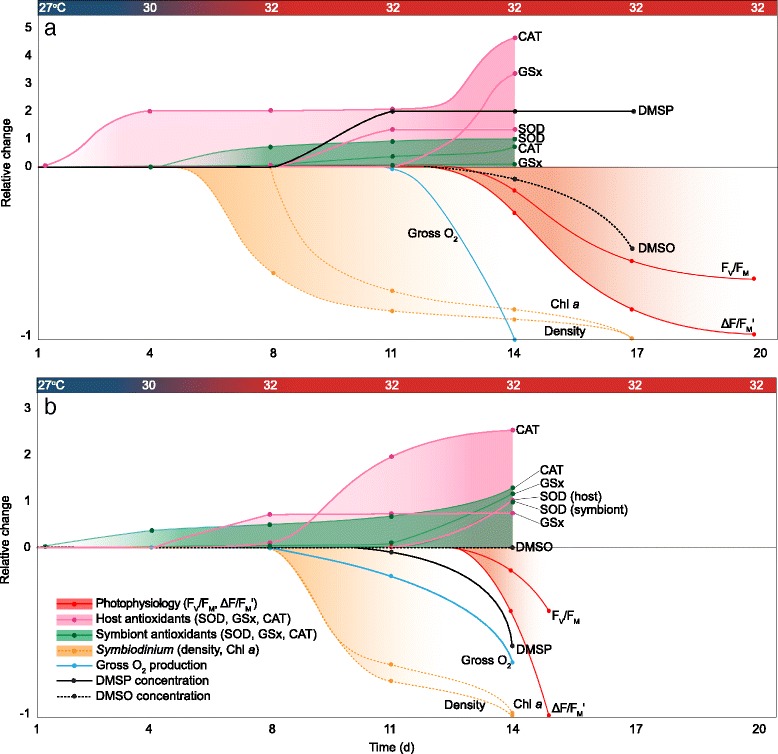
A time-resolved visualisation for the coral holobiont comparing the response of (**a**) *Acropora millepora* and (**b**) *Stylophora pistillata* to thermal stress over time (days). The graphical illustration of the changes to host and symbiont traits over time was constructed using Adobe Illustrator (CS6, Adobe Systems Software Ireland Ltd., CA, USA), where data points represent the magnitude of change (Fig. 4) for each trait that was significantly different from day 1 and the control at each time point (according to the PERMANOVA output). Lines are hand drawn interpolations of the change between time points with shading used to group host and symbiont responses to assist with interpretation. Photophysiological parameters (red solid lines and shading), host (pink solid lines and shading) and symbiont (green solid lines and shading) antioxidants (superoxide dismutase (SOD), glutathione (GSx) and catalase (CAT)/CAT-like activity), *Symbiodinium* pigmentation (including *Symbiodinium* density and chlorophyll *a*; orange dotted lines and shading), gross photosynthesis (blue solid line), dimethylsulfoniopropionate (DMSP) concentration (black solid line) and dimethyl sulfoxide (DMSO; black dotted line) are shown. The relative change is indicated by the left y-axis, note the change in scale for *S. pistillata* (**b**), where maximum relative change is 3. The time axis extends to day 20 for *A. millepora* because maximum/effective quantum yields were measured until day 20, whereas *S. pistillata* was more sensitive to thermal stress and measurements were not detected past day 15

When visualised over time, similarities and differences in the bleaching response between the two species are emphasised (Fig. 5). Onset of bleaching, represented as a decline in the *Symbiodinium* density, occurred after day 4 in *A. millepora* once water temperatures had reached beyond 30 °C and after day 8 for *S. pistillata* after temperatures had reached 32 °C. Prior to cell loss, changes to a few traits of both the host and symbiont became apparent in both species. In *A. millepora*, the antioxidant response was initiated in the host followed by the symbiont. In contrast, in *S. pistillata*, it was the symbiont that was the first to respond followed by the host on day 8. Post significant symbiont loss (> 50% decline) major pathophysiological responses occurred in both species across multiple traits. In *A. millepora*, remaining antioxidants of both the host and symbiont responded and DMSP increased. In *S. pistillata*, we saw an increased thermal stress response specifically driven by host CAT and a decline in gross photosynthesis. By day 14, both corals boosted their antioxidant activity closely corresponding to the onset of a measureable decline in symbiont photophysiological health. Beyond this climactic pathophysiological response there was severe loss of photosynthetic function and symbiont density was reduced to less than 5%.

## Discussion

Herein, we measured 14 stress-response indicators in two common corals from the Great Barrier Reef, *A. millepora* and *S. pistillata*, over 17 days of thermal stress. This revealed in detail the time-course of the bleaching process, including species-specific pathophysiologies in response to elevated temperature (Fig. 5). Importantly, the time-course revealed a well-defined cascade of conserved responses towards the bleached state. In both species, the initial onset of *Symbiodinium* expulsion preceded any severe pathophysiological responses of the host or symbiont and photophysiological health only declined in the last stages immediately prior to complete bleaching (Fig. 5). The cascade described here demarks a previously undefined non-linearity in the bleaching process, from the initial adjustments in the corals’ antioxidant systems, to the dramatic response correlated with a higher rate of bleaching and reduction in symbiont function. Using a systems approach, this newly described response-pattern, may provide important clues to the mechanism behind coral bleaching under elevated temperatures and the tolerance and sensitivity of various coral species.

### Bleaching susceptibility is largely independent of photosynthetic performance

Thermal stress caused a decline in the photosynthetic performance of the symbionts, indicative of accumulating photodamage [51–53]. However, the decline in photosynthetic health occurred after symbiont densities had decreased by more than 70%, a response that has been observed in previous studies [51, 54, 55], suggesting a decoupling of symbiont photodamage from the expulsion process. While reduced photosynthetic health is likely to result in expulsion as part of the general ‘house-hold’ functions in corals [56], our results add to the increasing pool of evidence that symbiont photosystem damage is not the initial driver of bleaching [33].

The earlier onset of expulsion in *A. millepora*, which harbours twice the number of symbiont cells per unit area than *S. pistillata*, supports previous work showing that bleaching susceptibility increases with the *Symbiodinium* density in the tissue [57] – where higher symbiont densities are proposed to result in enhanced accumulation of ROS in the host tissue and therefore elicit an earlier bleaching response. However, other explanations are also possible, including the effect of differences in the thermal tolerance of the respective symbiont clades [15, 58], or differences in host regulation of symbiont density and acquisition between the two coral species – *A. millepora* is a horizontal transmitter with greater flexibility in host-symbiont regulation and may readily expel and take up symbionts on a daily basis, whereas *S. pistillata* is a vertical transmitter with tight specific host-symbiont associations [59, 60] and may therefore be more reluctant to release its symbionts. This difference in maternal provisioning may also explain the earlier regulation of antioxidant activity in the symbionts of *S. pistillata*. As a vertical transmitter, it may have an innate strategy for thermal acclimation through regulating its antioxidants, rather than attempting to regulate symbiont density via expulsion. In contrast, the flexibility in symbiont association of *A. millepora* may preclude metabolic changes during early warming.

### Antioxidant response is species specific

In a recent study on thermal stress in corals, the observed antioxidant response was limited to an increase in host CAT activity [55], leading to the conclusion that the host response was independent from that of the symbiont. Apart from supporting an important role of CAT in the host response, these data contrast with the present study, where we found increases in host SOD and GSx as well as CAT activity in both coral species and, importantly, variations in the timing and magnitude of these antioxidant responses (Fig. 5). In *A. millepora* the antioxidant cascade started with an early increase in host CAT, followed by symbiont SOD, whereas, for *S. pistillata*, an early response in symbiont CAT-like activity was succeeded by host GSx. In both cases, however, host CAT showed the greatest relative increase under thermal stress. Additionally, the earlier study found no evidence of symbiont antioxidant activity in corals under thermal stress [55], again contrasting with our findings, which showed significant increases in symbiont SOD and CAT-like activity for *A. millepora* (hosting clade C3 symbionts) and symbiont SOD, GSx and CAT-like activity in clade C8a hosted by *S. pistillata*. One of the main differences between the symbiont antioxidant responses was the increase of GSx in the symbionts of *S. pistillata*, but not in *A. millepora*. Processes downstream from SOD, such as the glutathione system, have been shown to differ in activity between the most tolerant and susceptible types of *Symbiodinium*, despite similar responses in SOD activity [61], suggesting that the symbionts of *S. pistillata* could be more thermally tolerant, as indicated by the later bleaching response. Taken together, the difference in timing of bleaching and symbiont antioxidant response in our study could indicate either differences in host-driven defences or an important role of clade-dependent thermal-induced malfunction.

The ‘oxidative theory of coral bleaching’ proposes that the host is challenged by symbiont-generated H_2_O_2_ diffusing from the symbiont into the coral cytoplasm [62]. If, however, the symbiont represented a significant diffusive source of H_2_O_2_, it could be argued that an equal or greater antioxidant response would be expected in the symbiont, which was not the case here. In fact, under thermal stress, CAT activity was higher in the host than the symbiont, a response also shown previously [55, 63, 64], suggesting that the host’s scavenging capacity is likely capable of suppressing any symbiont-derived H_2_O_2_. However, a recent study showed that symbiont gene expression rarely exceeds a two-fold change [65], and therefore it cannot be ruled out that the symbiont has a limited capacity to upregulate its antioxidants, thus explaining the lower response measured. Nevertheless, if photo-oxidative stress in the symbiont is the primary event in the bleaching cascade, as previously suggested [6, 51], it could also be assumed that a decline in photosynthetic function would be visible initially in the symbiont prior to significant antioxidant upregulation and expulsion, a result our data do not support. Therefore, while verifying an increase in oxidative pressure during thermal stress, our results, along with an increasing pool of similar studies, are not congruent with the idea of accumulation of symbiont-derived free radicals as the initial cause of bleaching [55, 66] and instead suggest species-specific differences, where the initial pathophysiological response is largely independent of symbiont photosynthetic dysfunction.

### Late stage bleaching may be linked to ROS production

By measuring multiple traits over time, we found a strong correlation between photosystem collapse in the symbiont and an additional, secondary boost in the symbiont and host antioxidants (Fig. 5). This secondary response could indeed be explained by the oxidative theory of coral bleaching, whereby increased ROS production by the symbiont, from photosystem failure, results in H_2_O_2_ leaching into the host, and in this case, causes a boost in the antioxidants for H_2_O_2_ removal. As such, while our study reveals differences in the timing and magnitude of the antioxidant response for both species, the parallels in this late stage response support previous findings of a connection between symbiont stress and host antioxidant regulation [30, 55]. The delay in this response (days after the onset of symbiont expulsion), however, highlights the potential inaccuracy with using symbiont photosystem health as a proxy for the onset of thermal bleaching, and suggests that studies focussed solely on this trait as a cue for bleaching have potentially biased our understanding of coral bleaching towards mechanisms that are mainly active during the more severe stage of the bleaching process.

### DMSP as an early biomarker for bleaching is species specific

The most striking difference between the coral species in response to thermal stress was the divergence of temperature-induced changes in DMSP concentrations. The disparity in initial concentrations of DMSP for *A. millepora* (11 nmol/mm^2^) and *S. pistillata* (2 nmol/mm^2^) cannot be solely explained by differences in initial symbiont densities and could therefore be attributed to differences in cladal DMSP production rates. However, given the sustained production of DMSP in *A. millepora* even after substantial loss of symbionts, it is more likely due to the additional contribution of DMSP by the animal host in *A. millepora*, as previously shown by Raina et al. [26], or by the associated microbial community [26, 27]. In contrast, the decline in DMSP concentration with cell loss in *S. pistillata* indicates that DMSP production might only be *Symbiodinium* derived in this species. Taking into account these differences, it is evident that the role of DMSP is not equal in the two species of corals and therefore should be evaluated on a species-specific basis.

The potential importance of DMSP in the coral holobiont is a result of its ability to scavenge OH^•^ radicals and thereby functions as an antioxidant when present in high concentrations [24]. Indeed, previous studies have linked increases in DMSO, the oxidation product of DMSP, to enhanced protection from oxidative stress in corals [54]. If DMSP production in *S. pistillata* is solely *Symbiodinium* derived, then the observed increase in DMSP and DMSO per *Symbiodinium* cell under thermal stress would corroborate its role as an antioxidant in the symbionts. However, in the case of *A. millepora*, the story is not as clear – while DMSP concentrations increased with increasing host SOD (suggesting a link with oxidative stress), the lack of subsequent changes over time suggests that DMSP production is not directly related to the absolute level of ROS pressure, but is rather switched on at a certain degree of stress and maintained for the duration of that stress. This lack of connection between DMSP concentration and relative ROS pressure is further supported by the lack of accumulation of DMSO, which would be expected if DMSP was oxidised at a significant rate. Because of its strong response in *A. millepora*, we propose that DMSP is a useful early biomarker of thermal (and other types of physiological) stress, with the potential to apply this trait more broadly to the *Acropora* genus.

## Conclusion

Our study emphasises the value of using a multi-trait systems approach over time to understand the bleaching response in corals. We identified a cascade of pathophysiological responses that were common to both taxa, and delivered an overview of the time-dependent key processes that occur during coral bleaching. We were able to ascertain that susceptibility to bleaching is independent of photosynthetic performance and that antioxidant activity precedes symbiont photoinhibition in these two species of coral. Our time-resolved study of multiple traits allowed us to identify a potential late stage interaction between symbiont and host oxidative stress in both corals, which may capture the hypothesised leaching of H_2_O_2_ from a compromised symbiont to an already thermally stressed host. Yet, given the lag-time between symbiont loss, combined host-symbiont antioxidant response and symbiont photosynthetic dysfunction, it seems likely that this late-stage response is uncoupled from the initiation of the bleaching process. We revealed that the timing and magnitude of the antioxidant and DMSP response to elevated temperatures varied between the two species, suggestive of unique strategies for acclimating to thermal anomalies, underlining the potential importance of maternal provisioning, host species and symbiont type in bleaching susceptibility.

## Additional file

Additional file 1: Supplementary methods, figures and tables. (DOCX 1685 kb)
