# Supplementary material for: A multi-trait systems approach reveals a response cascade to bleaching in corals
Source: BMC Biol. 2017 Dec 7;15:117. doi: 10.1186/s12915-017-0459-2 (PMC5719617; doi:10.1186/s12915-017-0459-2)
Supplement: Additional file 1: — Supplementary methods, figures and tables. (DOCX 1685 kb) [file 12915_2017_459_MOESM1_ESM.docx]

**SUPPORTING INFORMATION**

# Methods

*Symbiodinium diversity: PCR and sequence analyses*

PCR reagents included 1 × AmpliTaq Gold® Buffer (Life Technologies), 2 mM MgCl_2_, 0.25 μM dNTPs, 10 μg BSA, 5 pmol of each primer, 0.12 × SYBR® Green (Life Technologies), 1 Unit AmpliTaq Gold DNA polymerase (Life Technologies), 2 μL of DNA, and Ultrapure™Distilled Water (Life Technologies) made up to 25 μL. PCR was executed on an Applied Biosystems StepOnePlus Real-Time PCR system under the following conditions: initial denaturation at 95°C for 5 min, followed by 38 cycles of 30 sec at 95°C, 30 sec at 52°C, and 45 sec at 72°C, and completed with a final extension for 10 min at 72°C. Duplicates originating from each sample were combined prior to amplicon pooling and library preparation.

The amplicon library for sequencing was prepared by pooling PCR products into equimolar ratios based on qPCR and quantification using a Labchip® GX Touch HT (Perkin Elmer). To assess cross-contamination, PCR negative controls and amplification using the blank DNA extraction control as template were also included in the final library for sequencing. Amplicons in the library were size-selected using a Pippin Prep (Sage Science) and purified using the Qiaquick PCR Purification Kit (Qiagen). The volume of purified library added for sequencing was determined using a Labchip® GX Touch HT (Perkin Elmer), and sequenced using a 500 cycle MiSeq® v2 Reagent Kit and standard flow cell (2 × 250 paired end) on an Illumina MiSeq platform.

Only reads containing 100% identity matches to Illumina adaptor, index barcodes, and the ITSD and ITS2rev2 sequences identified using Geneious® 8.1.4 [1] were kept for downstream analyses. Mothur 1.36.1 [2] was used to remove singletons, sequences that had an average Q score ≤25, and reads that contained ambiguous bases. Potential chimeras were identified using Perseus [3] and removed from the dataset. To remove non-target DNA (eg. coral ITS2 data), amplicons were searched against the NCBI nt nucleotide database using blastn on the Magnus Cray XC40 system located at the Pawsey Supercomputing Centre at Technology Park, WA, and *Symbiodinium* sequences extracted using MEGAN 5.11.3 [4]. In Mothur, *Symbiodinium* sequence depth was normalised across samples by sub-sampling the reads to the lowest occurring number (n = 4764 per sample), and sorted into OTUs at a 97% similarity threshold for each clade separately, as in Stat et al. [5]. OTU representative sequences were identified by comparing against the Geosymbio database [6] and are available in GenBank (Accession numbers: KY825747-KY825767).

# References

1. Kearse M, Moir R, Wilson A, Stones-Havas S, Cheung M, Sturrock S, Buxton S, Cooper A, Markowitz S, Duran C *et al*. Geneious Basic: an integrated and extendable desktop software platform for the organization and analysis of sequence data. Bioinformatics. 2012; 28(12):1647-1649.

2. Schloss PD, Westcott SL, Ryabin T, Hall JR, Hartmann M, Hollister EB, Lesniewski RA, Oakley BB, Parks DH, Robinson CJ *et al*. Introducing mothur: open-source, platform-independent, community-supported software for describing and comparing microbial communities. Appl Environ Microbiol. 2009; 75(23):7537-7541.

3. Quince C, Lanzen A, Davenport RJ, Turnbaugh PJ. Removing noise from pyrosequenced amplicons. BMC Bioinformatics. 2011; 12:38.

4. Huson DH, Auch AF, Qi J, Schuster SC. MEGAN analysis of metagenomic data. Genome Res. 2007; 17(3):377-386.

5. Stat M, Yost DM, Gates RD. Geographic structure and host specificity shape the community composition of symbiotic dinoflagellates in corals from the Northwestern Hawaiian Islands. Coral Reefs. 2015; 34(4):1075-1086.

6. Franklin EC, Stat M, Pochon X, Putnam HM, Gates RD. GeoSymbio: a hybrid, cloud-based web application of global geospatial bioinformatics and ecoinformatics for Symbiodinium–host symbioses. Molecular Ecology Resources. 2012; 12:369-373.

# Supplementary tables

**Table S1.** Details of the two-factor univariate PERMANOVA test using a resemblance matrix based on Euclidean distance and factors as fixed effects (with time four-six levels and temperature two levels as fixed factors) for all variables analysed for *Acropora millepora*.

| Source of variation | *df* | SS | MS | Pseudo *F* | p(MC) |
| --- | --- | --- | --- | --- | --- |
| ***ΔF/F_M_' (Effective quantum yield)*** |  |  |  |  |  |
| Temperature | 1 | 1.2877 | 1.2877 | 334.430 | 0.0001 |
| Time | 18 | 2.2647 | 0.12582 | 32.676 | 0.0001 |
| Time × temperature | 18 | 2.2273 | 0.12374 | 32.136 | 0.0001 |
| Residual | 152 | 0.58528 | 3.85E-03 |  |  |
| Total | 189 | 6.3651 |  |  |  |
| ***F_V_/F_M_ (maximum quantum yield)*** |  |  |  |  |  |
| Temperature | 1 | 0.45925 | 0.45925 | 358.820 | 0.0001 |
| Time | 17 | 0.99517 | 5.85E-02 | 45.738 | 0.0001 |
| Time × temperature | 17 | 0.95482 | 5.62E-02 | 43.883 | 0.0001 |
| Residual | 144 | 0.1843 | 1.28E-03 |  |  |
| Total | 179 | 2.5935 |  |  |  |
| ***Symbiodinium density*** |  |  |  |  |  |
| Temperature | 1 | 4.20E+12 | 4.20E+12 | 50.495 | 0.0001 |
| Time | 5 | 5.30E+12 | 1.06E+12 | 12.756 | 0.0001 |
| Time × temperature | 5 | 1.26E+12 | 2.52E+11 | 3.037 | 0.0190 |
| Residual | 48 | 3.99E+12 | 8.31E+10 |  |  |
| Total | 59 | 1.48E+13 |  |  |  |
| ***Chlorophyll a*** |  |  |  |  |  |
| Temperature | 1 | 1.11E+01 | 1.11E+01 | 16.717 | 0.0002 |
| Time | 5 | 1.42E+01 | 2.84E+00 | 4.288 | 0.0027 |
| Time × temperature | 5 | 1.36E+01 | 2.72E+00 | 4.102 | 0.0049 |
| Residual | 48 | 3.18E+01 | 6.63E-01 |  |  |
| Total | 59 | 7.07E+01 |  |  |  |
| ***Gross O_2_ production*** |  |  |  |  |  |
| Temperature | 1 | 9.11E-02 | 9.11E-02 | 12.561 | 0.0015 |
| Time | 3 | 0.43043 | 0.14348 | 19.788 | 0.0001 |
| Time × temperature | 3 | 0.38246 | 0.12749 | 17.583 | 0.0001 |
| Residual | 24 | 0.17401 | 7.25E-03 |  |  |
| Total | 31 | 1.078 |  |  |  |
| ***O_2_ flux (light)*** |  |  |  |  |  |
| Temperature | 1 | 1.69E-01 | 1.69E-01 | 40.253 | 0.0001 |
| Time | 3 | 0.32861 | 0.10954 | 26.150 | 0.0001 |
| Time × temperature | 3 | 0.23496 | 7.83E-02 | 18.698 | 0.0001 |
| Residual | 24 | 0.10053 | 4.19E-03 |  |  |
| Total | 31 | 0.8327 |  |  |  |
| ***O_2_ flux (dark)*** |  |  |  |  |  |
| Temperature | 1 | 9.75E-03 | 9.75E-03 | 7.625 | 0.0118 |
| Time | 3 | 2.80E-02 | 9.34E-03 | 7.306 | 0.0014 |
| Time × temperature | 3 | 2.60E-02 | 8.66E-03 | 6.775 | 0.0020 |
| Residual | 24 | 3.07E-02 | 1.28E-03 |  |  |
| Total | 31 | 9.44E-02 |  |  |  |
| ***Photosynthesis: respiration ratio*** |  |  |  |  |  |
| Temperature | 1 | 68.21 | 68.21 | 14.701 | 0.0004 |
| Time | 3 | 109.07 | 36.356 | 7.836 | 0.0010 |
| Time × temperature | 3 | 27.829 | 9.2764 | 1.999 | 0.1458 |
| Residual | 24 | 111.36 | 4.6398 |  |  |
| Total | 31 | 316.46 |  |  |  |
| ***DMSP (nmol)*** |  |  |  |  |  |
| Temperature | 1 | 8.80E+02 | 8.80E+02 | 31.691 | 0.0001 |
| Time | 5 | 1.72E+03 | 3.44E+02 | 12.402 | 0.0001 |
| Time × temperature | 5 | 1.04E+03 | 2.08E+02 | 7.487 | 0.0002 |
| Residual | 48 | 1.33E+03 | 2.78E+01 |  |  |
| Total | 59 | 4.97E+03 |  |  |  |
| ***DMSP (per cell)*** |  |  |  |  |  |
| Temperature | 1 | 6.49E+07 | 6.49E+07 | 18.230 | 0.0001 |
| Time | 5 | 1.96E+08 | 3.92E+07 | 10.998 | 0.0001 |
| Time × temperature | 5 | 1.90E+08 | 3.81E+07 | 10.696 | 0.0001 |
| Residual | 48 | 1.71E+08 | 3.56E+06 |  |  |
| Total | 59 | 6.22E+08 |  |  |  |
| ***DMSO (nmol)*** |  |  |  |  |  |
| Temperature | 1 | 1793.5 | 1793.5 | 19.541 | 0.0002 |
| Time | 5 | 1475.7 | 295.15 | 3.216 | 0.0156 |
| Time × temperature | 5 | 1022 | 204.41 | 2.227 | 0.0696 |
| Residual | 48 | 4405.6 | 91.783 |  |  |
| Total | 59 | 8696.9 |  |  |  |
| ***DMSO (per cell)*** |  |  |  |  |  |
| Temperature | 1 | 3.83E+06 | 3.83E+06 | 15.897 | 0.0003 |
| Time | 5 | 5.78E+06 | 1.16E+06 | 4.796 | 0.0014 |
| Time × temperature | 5 | 5.38E+06 | 1.08E+06 | 4.459 | 0.0027 |
| Residual | 48 | 1.16E+07 | 2.41E+05 |  |  |
| Total | 59 | 2.66E+07 |  |  |  |
| ***Host superoxide dismutase*** |  |  |  |  |  |
| Temperature | 1 | 303.11 | 303.11 | 2.370 | 0.1289 |
| Time | 4 | 3719.9 | 929.97 | 7.272 | 0.0004 |
| Time × temperature | 4 | 1214.1 | 303.52 | 2.373 | 0.0705 |
| Residual | 40 | 5115.6 | 127.89 |  |  |
| Total | 49 | 10353 |  |  |  |
| ***Algae superoxide dismutase*** |  |  |  |  |  |
| Temperature | 1 | 2836.9 | 2836.9 | 17.449 | 0.0004 |
| Time | 4 | 1701.7 | 425.42 | 2.617 | 0.0527 |
| Time × temperature | 4 | 2625.7 | 656.43 | 4.038 | 0.0089 |
| Residual | 40 | 6503.4 | 162.58 |  |  |
| Total | 49 | 13668 |  |  |  |
| ***Superoxide dismutase (per cell)*** |  |  |  |  |  |
| Temperature | 1 | 1082.9 | 1082.9 | 2.113 | 0.1512 |
| Time | 4 | 3816.5 | 954.13 | 1.862 | 0.1340 |
| Time × temperature | 4 | 2300.7 | 575.17 | 1.122 | 0.3504 |
| Residual | 40 | 20498 | 512.45 |  |  |
| Total | 49 | 27698 |  |  |  |
| ***Host glutathione*** |  |  |  |  |  |
| Temperature | 1 | 1.11E+06 | 1.11E+06 | 19.883 | 0.0002 |
| Time | 4 | 2.00E+06 | 5.00E+05 | 8.959 | 0.0001 |
| Time × temperature | 4 | 1.99E+06 | 4.96E+05 | 8.901 | 0.0001 |
| Residual | 40 | 2.23E+06 | 55765 |  |  |
| Total | 49 | 7.32E+06 |  |  |  |
| ***Algae glutathione*** |  |  |  |  |  |
| Temperature | 1 | 858.37 | 858.37 | 2.372 | 0.1297 |
| Time | 4 | 2448.1 | 612.02 | 1.691 | 0.1751 |
| Time × temperature | 4 | 964.14 | 241.03 | 0.666 | 0.6178 |
| Residual | 40 | 14478 | 361.94 |  |  |
| Total | 49 | 18748 |  |  |  |
| ***Glutathione (per cell)*** |  |  |  |  |  |
| Temperature | 1 | 688.7 | 688.7 | 2.271 | 0.1424 |
| Time | 4 | 2782 | 695.51 | 2.293 | 0.0818 |
| Time × temperature | 4 | 815.94 | 203.98 | 0.673 | 0.6224 |
| Residual | 40 | 12131 | 303.27 |  |  |
| Total | 49 | 16417 |  |  |  |
| ***Host catalase*** |  |  |  |  |  |
| Temperature | 1 | 14732 | 14732 | 64.191 | 0.0001 |
| Time | 4 | 7389 | 1847.3 | 8.049 | 0.0001 |
| Time × temperature | 4 | 6602.9 | 1650.7 | 7.193 | 0.0002 |
| Residual | 40 | 9179.9 | 229.5 |  |  |
| Total | 49 | 37904 |  |  |  |
| ***Algae catalase-like activity*** |  |  |  |  |  |
| Temperature | 1 | 382.59 | 382.59 | 10.911 | 0.0027 |
| Time | 4 | 483.27 | 120.82 | 3.446 | 0.0174 |
| Time × temperature | 4 | 347.31 | 86.828 | 2.476 | 0.0601 |
| Residual | 40 | 1402.5 | 35.064 |  |  |
| Total | 49 | 2615.7 |  |  |  |
| ***Catalase-like activity (per cell)*** |  |  |  |  |  |
| Temperature | 1 | 1123 | 1123 | 16.942 | 0.0001 |
| Time | 4 | 1856.7 | 464.19 | 7.003 | 0.0001 |
| Time × temperature | 4 | 1361.4 | 340.35 | 5.134 | 0.0016 |
| Residual | 40 | 2651.5 | 66.288 |  |  |
| Total | 49 | 6992.7 |  |  |  |

**Table S2.** Details of the two-factor univariate PERMANOVA test using a resemblance matrix based on Euclidean distance and factors as fixed effects (with time four-six levels and temperature two levels as fixed factors) for all variables analysed for *Stylophora pistillata*.

| Source of variation | *df* | SS | MS | Pseudo *F* | p(MC) |
| --- | --- | --- | --- | --- | --- |
| ***ΔF/F_M_' (Effective quantum yield)*** |  |  |  |  |  |
| Temperature | 1 | 2.0657 | 2.0657 | 624.600 | 0.0001 |
| Time | 18 | 3.221 | 0.17895 | 54.108 | 0.0001 |
| Time × temperature | 18 | 2.8453 | 0.15807 | 47.797 | 0.0001 |
| Residual | 152 | 0.50269 | 3.31E-03 |  |  |
| Total | 189 | 8.6347 |  |  |  |
| ***F_V_/F_M_ (maximum quantum yield)*** |  |  |  |  |  |
| Temperature | 1 | 1.1515 | 1.1515 | 558.670 | 0.0001 |
| Time | 17 | 2.4321 | 0.14306 | 69.409 | 0.0001 |
| Time × temperature | 17 | 2.5715 | 0.15127 | 73.389 | 0.0001 |
| Residual | 144 | 0.29681 | 2.06E-03 |  |  |
| Total | 179 | 6.4519 |  |  |  |
| ***Symbiodinium density*** |  |  |  |  |  |
| Temperature | 1 | 1.32E+11 | 1.32E+11 | 6.218 | 0.0169 |
| Time | 4 | 4.81E+11 | 1.20E+11 | 5.656 | 0.0006 |
| Time × temperature | 4 | 2.36E+11 | 5.90E+10 | 2.776 | 0.0384 |
| Residual | 40 | 8.51E+11 | 2.13E+10 |  |  |
| Total | 49 | 1.70E+12 |  |  |  |
| ***Chlorophyll a*** |  |  |  |  |  |
| Temperature | 1 | 1.5123 | 1.5123 | 19.019 | 0.0001 |
| Time | 4 | 1.7524 | 0.43809 | 5.510 | 0.0019 |
| Time × temperature | 4 | 1.8556 | 0.4639 | 5.834 | 0.0011 |
| Residual | 40 | 3.1805 | 7.95E-02 |  |  |
| Total | 49 | 8.3007 |  |  |  |
| ***Gross O_2_ production*** |  |  |  |  |  |
| Temperature | 1 | 9.56E-02 | 9.56E-02 | 12.562 | 0.0020 |
| Time | 3 | 0.27665 | 9.22E-02 | 12.112 | 0.0001 |
| Time × temperature | 3 | 0.11153 | 3.72E-02 | 4.883 | 0.0087 |
| Residual | 24 | 0.18272 | 7.61E-03 |  |  |
| Total | 31 | 0.66654 |  |  |  |
| ***O_2_ flux (light)*** |  |  |  |  |  |
| Temperature | 1 | 1.22E-01 | 1.22E-01 | 26.992 | 0.0001 |
| Time | 3 | 1.71E-01 | 5.69E-02 | 12.618 | 0.0001 |
| Time × temperature | 3 | 1.10E-01 | 3.66E-02 | 8.123 | 0.0008 |
| Residual | 24 | 1.08E-01 | 4.51E-03 |  |  |
| Total | 31 | 5.11E-01 |  |  |  |
| ***O_2_ flux (dark)*** |  |  |  |  |  |
| Temperature | 1 | 4.35E-03 | 4.35E-03 | 4.424 | 0.0443 |
| Time | 3 | 2.18E-02 | 7.27E-03 | 7.386 | 0.0007 |
| Time × temperature | 3 | 6.22E-03 | 2.07E-03 | 2.107 | 0.1286 |
| Residual | 24 | 2.36E-02 | 9.84E-04 |  |  |
| Total | 31 | 5.60E-02 |  |  |  |
| ***Photosynthesis: respiration ratio*** |  |  |  |  |  |
| Temperature | 1 | 2.20E+01 | 2.20E+01 | 59.049 | 0.0001 |
| Time | 3 | 5.0475 | 1.68E+00 | 4.526 | 0.0112 |
| Time × temperature | 3 | 10.415 | 3.47E+00 | 9.339 | 0.0001 |
| Residual | 24 | 8.9214 | 3.72E-01 |  |  |
| Total | 31 | 46.334 |  |  |  |
| ***DMSP (nmol)*** |  |  |  |  |  |
| Temperature | 1 | 8.0447 | 8.0447 | 22.529 | 0.0002 |
| Time | 4 | 4.4624 | 1.1156 | 3.124 | 0.0262 |
| Time × temperature | 4 | 10.936 | 2.734 | 7.657 | 0.0002 |
| Residual | 40 | 14.283 | 0.35708 |  |  |
| Total | 49 | 37.726 |  |  |  |
| ***DMSP (per cell)*** |  |  |  |  |  |
| Temperature | 1 | 18852 | 18852 | 8.323 | 0.0066 |
| Time | 4 | 92944 | 23236 | 10.258 | 0.0001 |
| Time × temperature | 4 | 37370 | 9342.4 | 4.125 | 0.0080 |
| Residual | 40 | 90603 | 2265.1 |  |  |
| Total | 49 | 2.40E+05 |  |  |  |
| ***DMSO (nmol)*** |  |  |  |  |  |
| Temperature | 1 | 4.70E-03 | 4.70E-03 | 0.328 | 0.5691 |
| Time | 4 | 0.10025 | 2.51E-02 | 1.749 | 0.1551 |
| Time × temperature | 4 | 5.84E-02 | 1.46E-02 | 1.018 | 0.4143 |
| Residual | 40 | 0.57319 | 1.43E-02 |  |  |
| Total | 49 | 0.7365 |  |  |  |
| ***DMSO (per cell)*** |  |  |  |  |  |
| Temperature | 1 | 3788.3 | 3788.3 | 17.398 | 0.0006 |
| Time | 4 | 9828.7 | 2457.2 | 11.285 | 0.0001 |
| Time × temperature | 4 | 9304 | 2326 | 10.683 | 0.0001 |
| Residual | 40 | 8709.5 | 217.74 |  |  |
| Total | 49 | 31630 |  |  |  |
| ***Host superoxide dismutase*** |  |  |  |  |  |
| Temperature | 1 | 368.61 | 368.61 | 46.845 | 0.0001 |
| Time | 4 | 363.46 | 90.864 | 11.547 | 0.0001 |
| Time × temperature | 4 | 174.03 | 43.508 | 5.529 | 0.0014 |
| Residual | 40 | 314.75 | 7.8687 |  |  |
| Total | 49 | 1220.8 |  |  |  |
| ***Algae superoxide dismutase*** |  |  |  |  |  |
| Temperature | 1 | 209.2 | 209.2 | 9.083 | 0.0042 |
| Time | 4 | 93.913 | 23.478 | 1.019 | 0.4206 |
| Time × temperature | 4 | 216.05 | 54.013 | 2.345 | 0.0720 |
| Residual | 40 | 921.29 | 23.032 |  |  |
| Total | 49 | 1440.5 |  |  |  |
| ***Superoxide dismutase (per cell)*** |  |  |  |  |  |
| Temperature | 1 | 264.67 | 264.67 | 15.103 | 0.0007 |
| Time | 4 | 597.81 | 149.45 | 8.528 | 0.0003 |
| Time × temperature | 4 | 544.74 | 136.18 | 7.771 | 0.0002 |
| Residual | 40 | 700.97 | 17.524 |  |  |
| Total | 49 | 2108.2 |  |  |  |
| ***Host glutathione*** |  |  |  |  |  |
| Temperature | 1 | 32978 | 32978 | 22.933 | 0.0001 |
| Time | 4 | 44372 | 11093 | 7.714 | 0.0001 |
| Time × temperature | 4 | 57636 | 14409 | 10.020 | 0.0001 |
| Residual | 40 | 57521 | 1438 |  |  |
| Total | 49 | 1.93E+05 |  |  |  |
| ***Algae glutathione*** |  |  |  |  |  |
| Temperature | 1 | 1660.6 | 1660.6 | 6.838 | 0.0142 |
| Time | 4 | 5215.1 | 1303.8 | 5.369 | 0.0015 |
| Time × temperature | 4 | 3491.8 | 872.95 | 3.595 | 0.0145 |
| Residual | 40 | 9713.9 | 242.85 |  |  |
| Total | 49 | 20081 |  |  |  |
| ***Glutathione (per cell)*** |  |  |  |  |  |
| Temperature | 1 | 25657 | 25657 | 16.136 | 0.0003 |
| Time | 4 | 1.80E+05 | 45033 | 28.322 | 0.0001 |
| Time × temperature | 4 | 1.41E+05 | 35175 | 22.122 | 0.0001 |
| Residual | 40 | 63602 | 1590.1 |  |  |
| Total | 49 | 4.10E+05 |  |  |  |
| ***Host catalase*** |  |  |  |  |  |
| Temperature | 1 | 5228 | 5228 | 17.292 | 0.0003 |
| Time | 4 | 3513.6 | 878.4 | 2.905 | 0.0351 |
| Time × temperature | 4 | 4712.3 | 1178.1 | 3.897 | 0.0095 |
| Residual | 40 | 12094 | 302.34 |  |  |
| Total | 49 | 25547 |  |  |  |
| ***Algae catalase-like activity*** |  |  |  |  |  |
| Temperature | 1 | 1630 | 1630 | 165.170 | 0.0001 |
| Time | 4 | 628.82 | 157.21 | 15.930 | 0.0001 |
| Time × temperature | 4 | 290.85 | 72.713 | 7.368 | 0.0003 |
| Residual | 40 | 394.74 | 9.8686 |  |  |
| Total | 49 | 2944.5 |  |  |  |
| ***Catalase-like activity (per cell)*** |  |  |  |  |  |
| Temperature | 1 | 3270.6 | 3270.6 | 23.602 | 0.0001 |
| Time | 4 | 6743.6 | 1685.9 | 12.166 | 0.0001 |
| Time × temperature | 4 | 5964.8 | 1491.2 | 10.761 | 0.0001 |
| Residual | 40 | 5542.9 | 138.57 |  |  |
| Total | 49 | 21522 |  |  |  |

# Supplementary figures

**
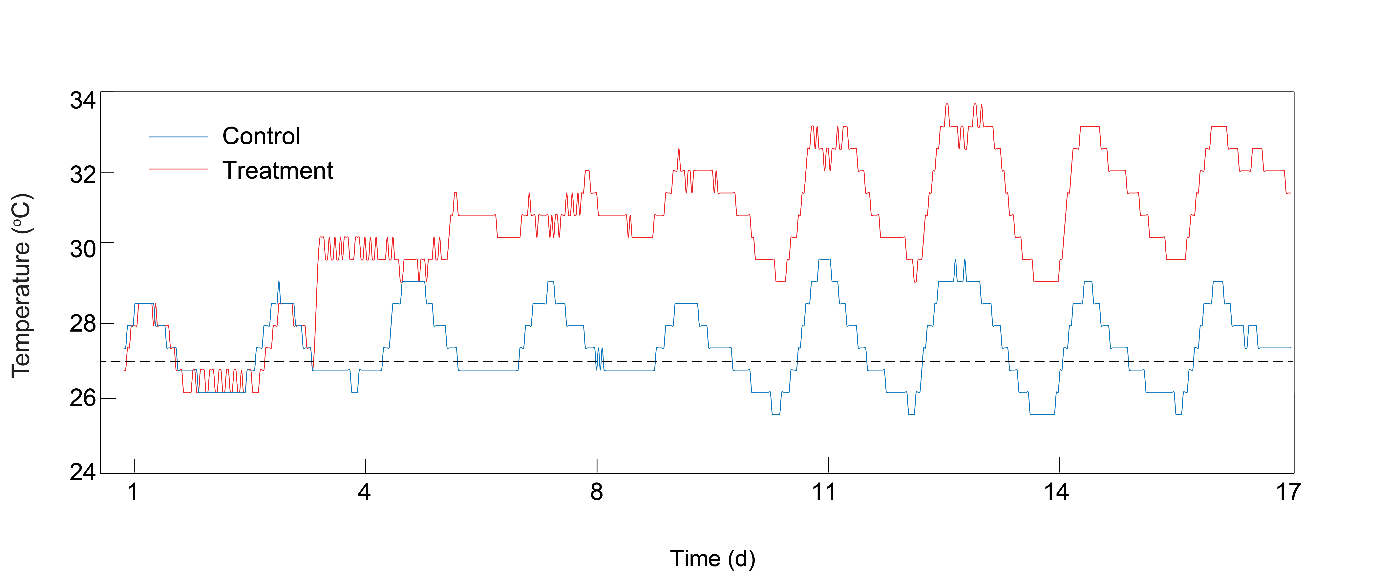
**

**Fig. S1** Temperature data from thermal stress experiment. Thermal ramp showing the control (blue lines) and treatment (red lines) including diel fluctuations. Temperatures were increased 1°C per day from 27°C (day 1) to 30°C (day 4) then 0.5°C per day to reach the target temperature of 32°C (day 8) and then held until day 14 for *Stylophora pistillata* and day 17 for *Acropora millepora*. The average temperature recorded for the control tank is indicated by the black dashed line.

#
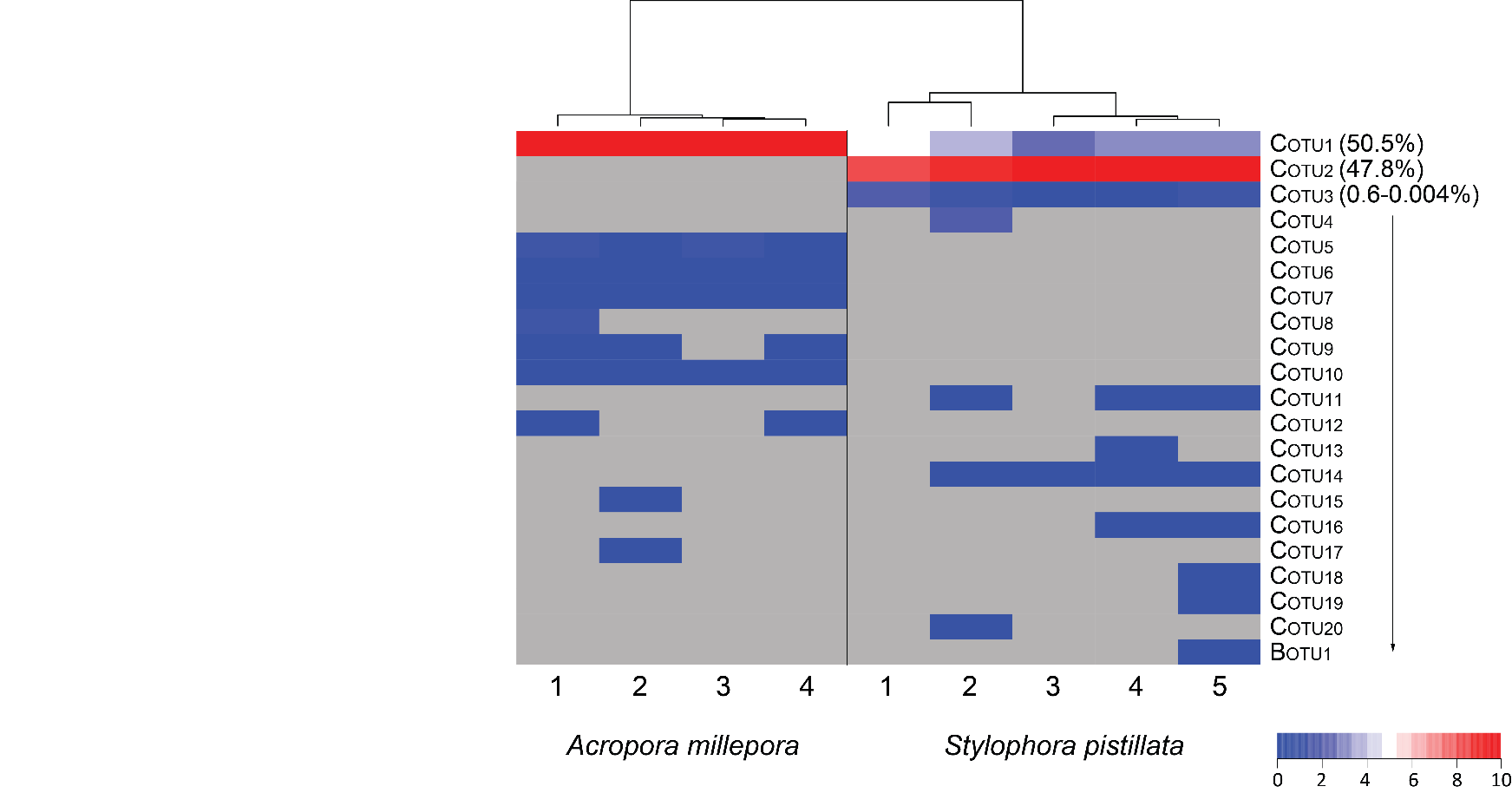


**Fig. S2** Heatmap of *Symbiodinium* OTUs in *Acropora millepora* and *Stylophora pistillata*. *Symbiodinium* ITS2 OTUs are displayed on the right, with the *Symbiodinium* clade that each OTU belongs to indicated by the initial capital letter. The overall frequency of the two most abundant OTUs is indicated in parentheses. All other OTUs occurred at a frequency between 0.6 - 0.004%. The colour (scale bar) represents the proportion of each OTU in the colony (after standardisation and square-root transformation), and grey boxes indicate an absence of the OTU.


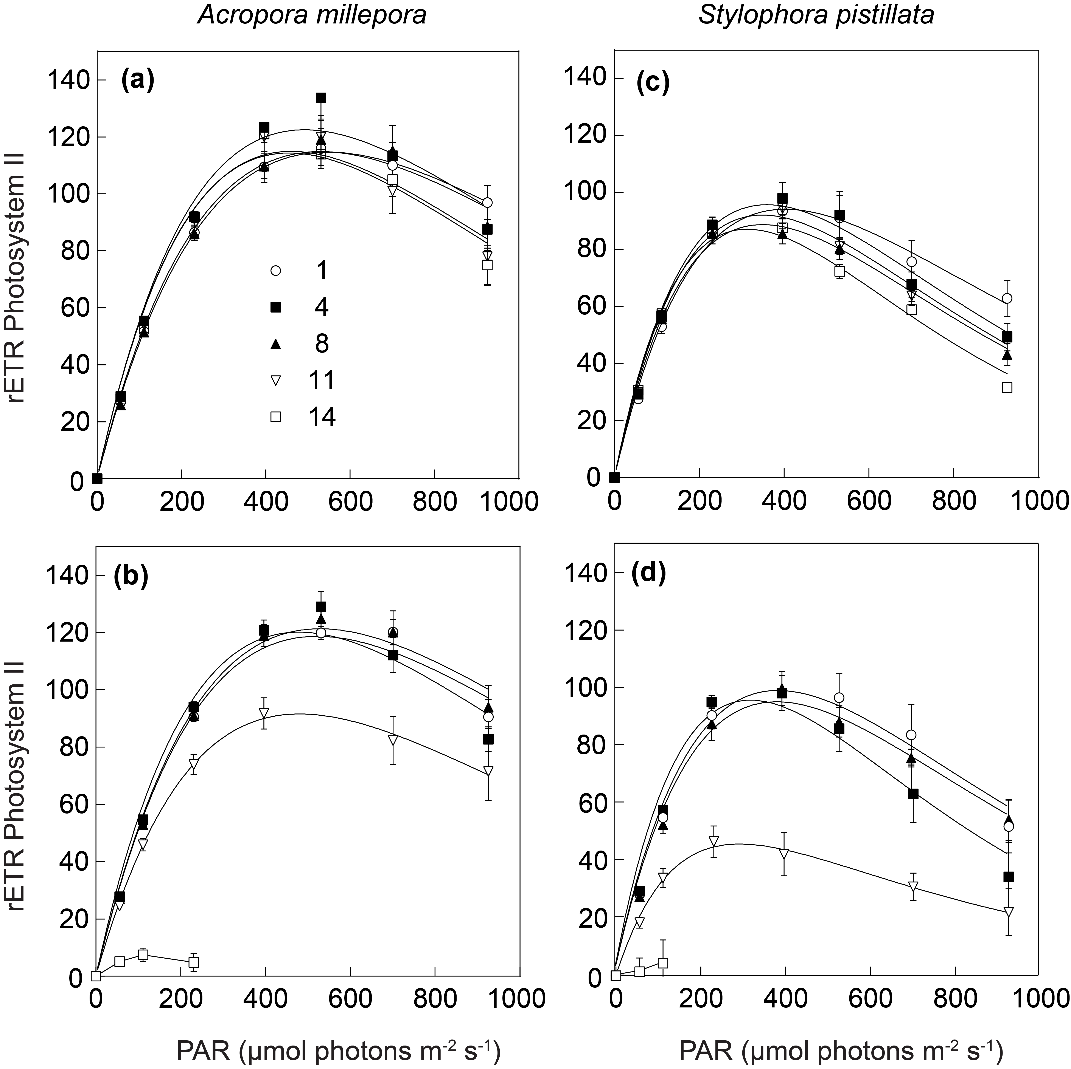


**Fig. S3** Relative electron transport rate (rETR) in photosystem II were calculated from conducting a seven-step steady state light curve using an Imaging PAM (Max/K, Walz GmbH, Effeltrich, Germany) for (a,b) *Acropora millepora* and (c,d) *Stylophora pistillata* for the control (27°C; a,c) and treatment (32°C; b,d) over each time point. Averages (± SE) shown (n = 5).


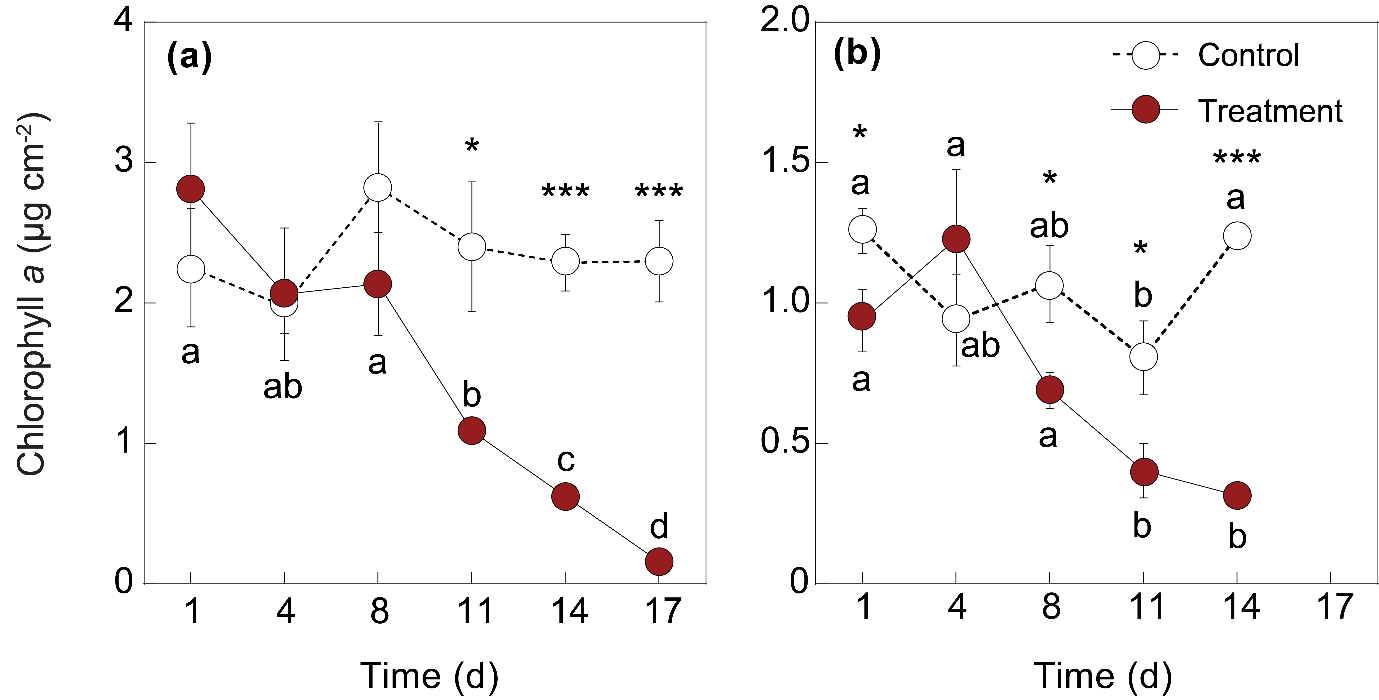


**Fig. S4** Chlorophyll *a* for (a) *Acropora millepora* and (b) *Stylophora pistillata* for the control (27°C; white circles, dashed line) and treatment (32°C; red circles, solid line) over time (days). Asterisks indicate significant differences between treatments where p < 0.05 (*) and < 0.001 (***) and letters indicate significant differences between time points for the heat-treated samples at *p* < 0.05. Averages (± SE) are shown (n = 5).


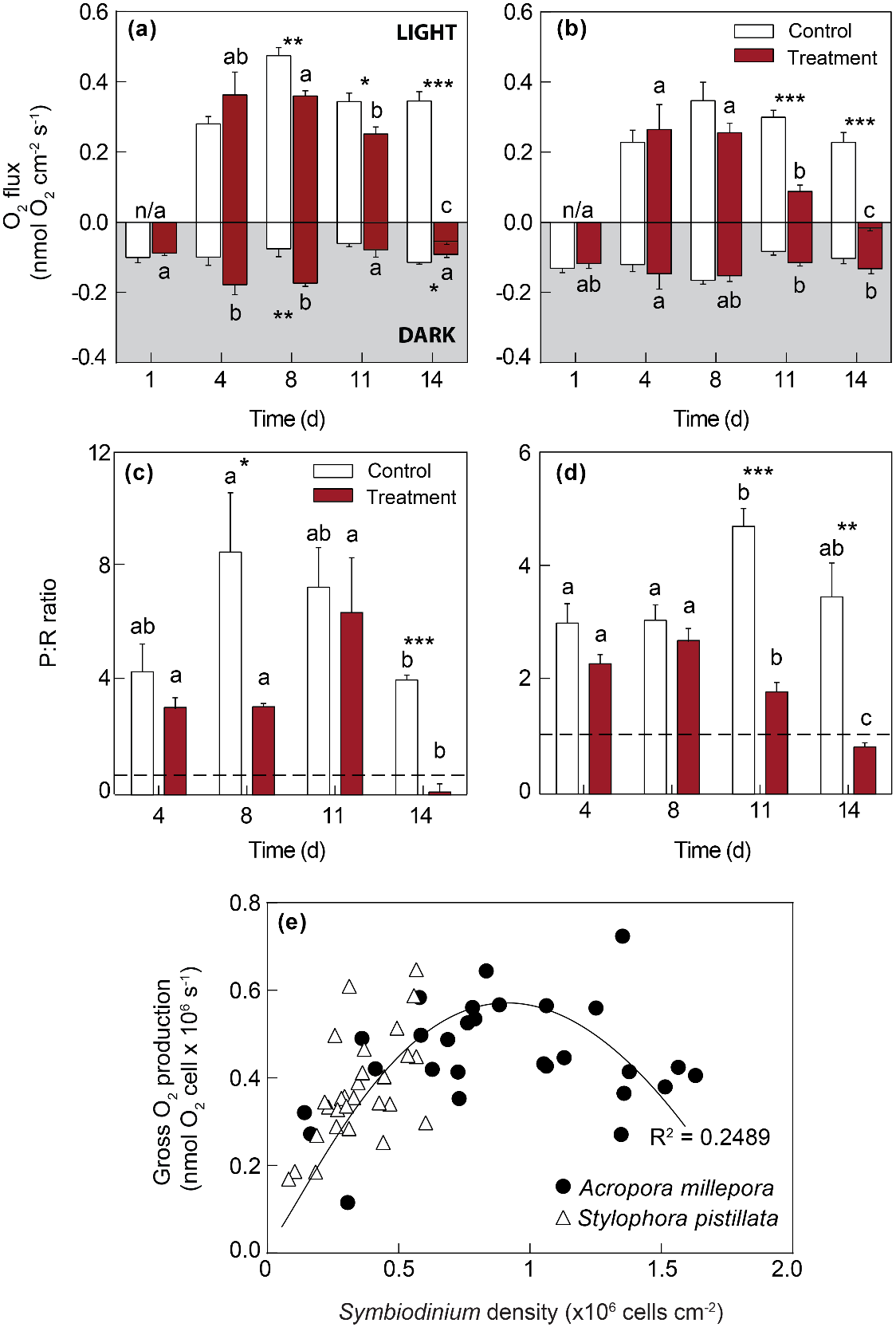


**Fig. S5** Oxygen flux for (a) *Acropora millepora* and (b) *Stylophora pistillata* for the control (27°C; white bars) and treatment (32°C; red bars) over 14 days. The bars above the solid line at zero represent the measurements in the light (holobiont photosynthesis) and below the line (grey shading) represent measurements in the dark (holobiont respiration). Photosynthesis: respiration ratio for (c) *Acropora millepora* and (d) *Stylophora pistillata* for the control (27°C; white bars) and treatment (32°C; red bars) over 14 days. The dashed line on the y-axis represents a P:R ratio of 1. Relationship between gross oxygen production and *Symbiodinium* density (e) for *Acropora millepora* (black circle symbols) and *Stylophora pistillata* (white triangle symbols). Asterisks indicate significant differences between treatments where p < 0.05 (*), < 0.01 (**) and < 0.001 (***) and n/a indicates data was not available for the respective time point. Letters indicate significant differences between time points for the heat-treated samples at *p* < 0.05. Averages (± SE) shown (n = 4).

#
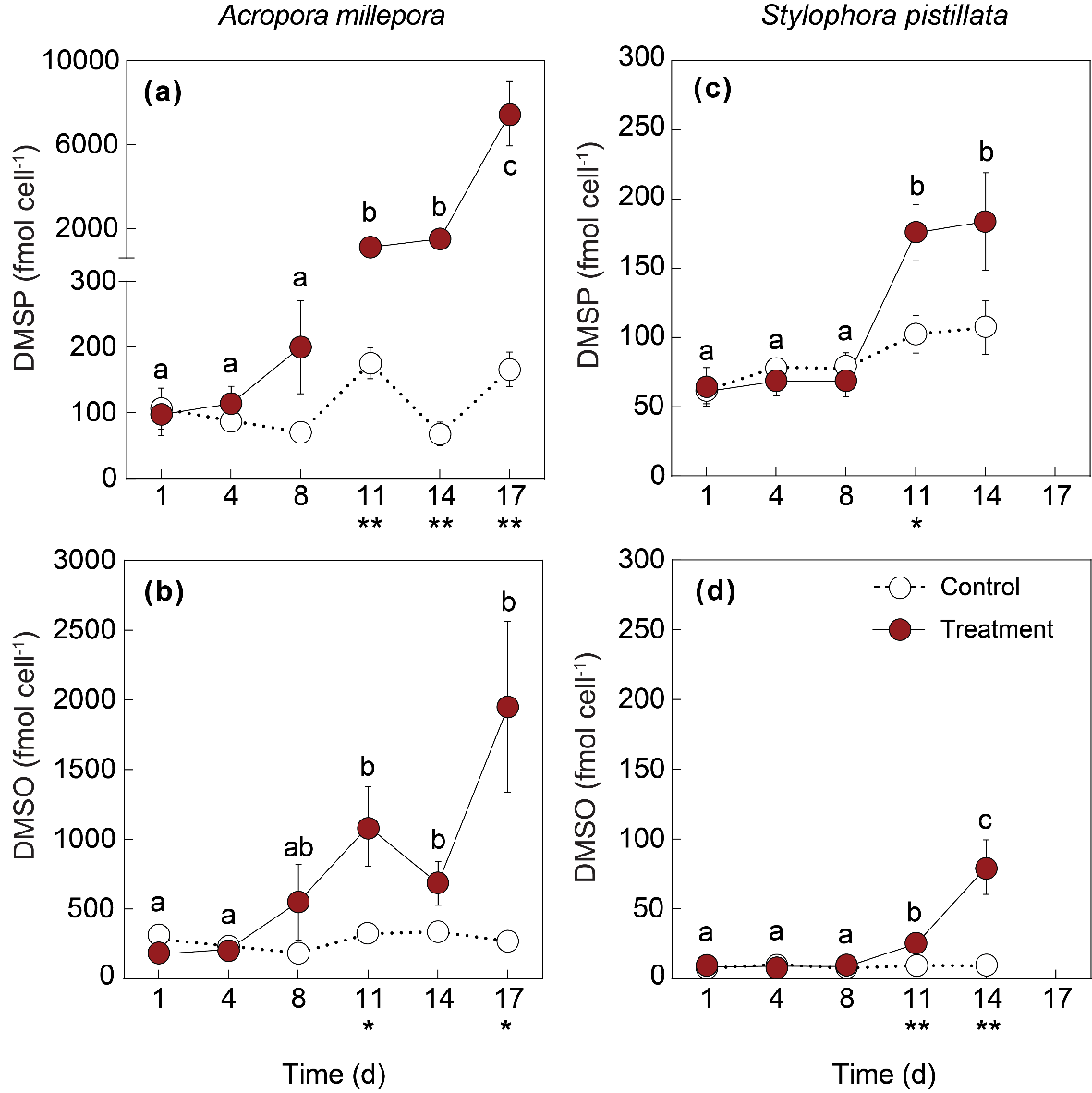


**Fig. S6** Concentrations of dimethylsulphoniopropionate (DMSP) and dimethylsulphoxide (DMSO) normalised to *Symbiodinium* cell density for (a,b) *Acropora millepora* and (c,d) *Stylophora pistillata* for the control (27°C; white circles, dashed line) and treatments (32°C; red circles, solid line) over time (days). Asterisks indicate significant differences between treatments where p < 0.05 (*) and < 0.01 (**) and letters indicate significant differences between time points for the heat-treated samples at *p* < 0.05. Averages (± SE) are shown (n = 5).


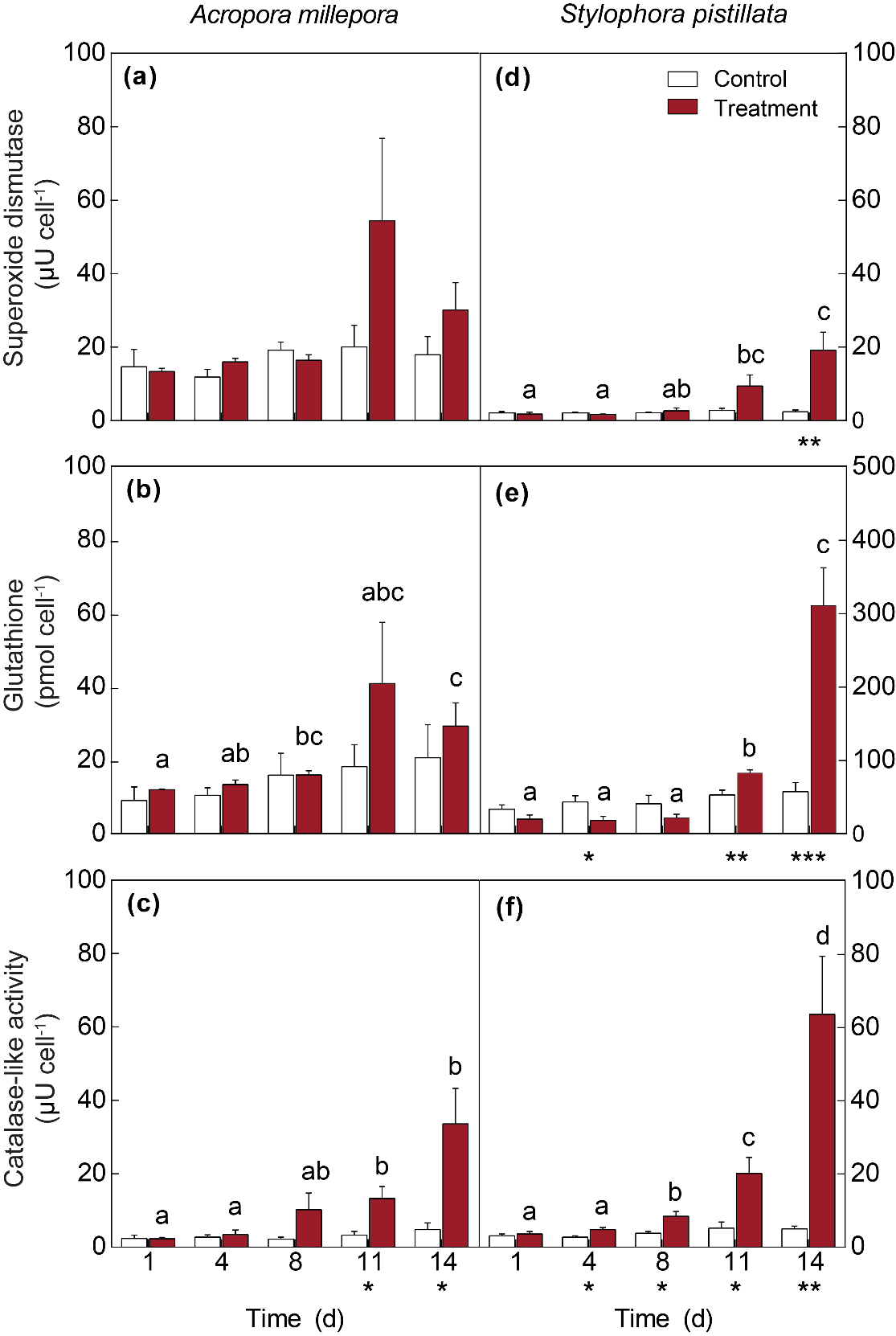


**Fig. S7** Symbiont antioxidant activity normalised to cell density for (a-c) *Acropora millepora* and (d-f) *Stylophora pistillata* showing superoxide dismutase, glutathione and catalase-like activity for the control (27°C; white bars) and treatments (32°C; red bars) over time. Asterisks indicate significant differences between treatments where p < 0.05 (*), < 0.01 (**) and < 0.001 (***) and letters indicate significant differences between time for the heat-treated samples at *p* < 0.05. Averages (± SE) are shown (n = 4-5).


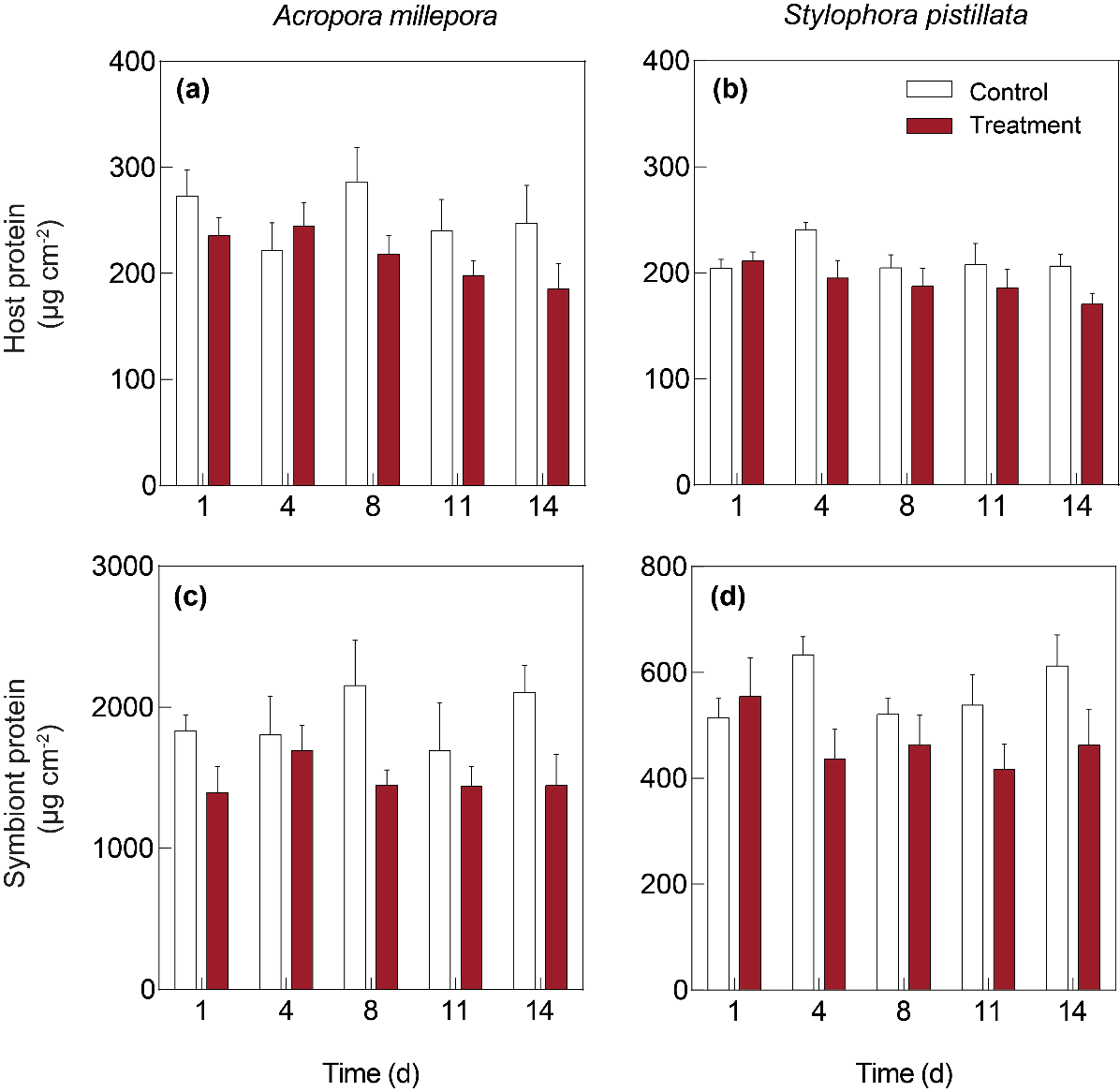


**Fig. S8** Host and symbiont protein content normalised to surface area for (a, c) *Acropora millepora* and (b, d) *Stylophora pistillata* for the control (27°C; white bars) and treatments (32°C; red bars) over time, showing no significant differences at *p* < 0.05. Averages (± SE) are shown (n = 5).


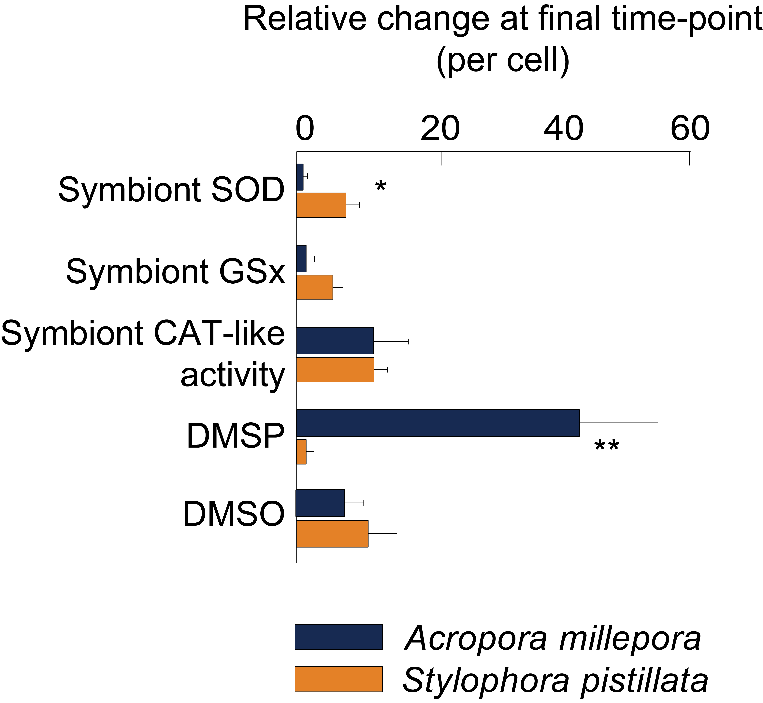


**Fig. S9** Comparison showing the relative change between day 1 and day 14 of the treatments for symbiont antioxidants activity for superoxide dismutase (SOD), glutathione (GSx), catalase-like activity (CAT), DMSP (dimethylsulphoniopropionate) and DMSO (dimethylsulphoxide) concentrations at 32°C in *Acropora millepora* (dark blue bars) and *Stylophora pistillata* (orange bars) normalised to *Symbiodinium*  density. Calculated as the relative change at the final time-point between the control and the treatment (X_treatment_ – X_control­ /_ X_control­_). Asterisk indicate significant differences between species at p < 0.05 (*) and < 0.01 (**).
